# Loss of Glycosaminoglycan Receptor Binding after Mosquito Cell Passage Reduces Chikungunya Virus Infectivity

**DOI:** 10.1371/journal.pntd.0004139

**Published:** 2015-10-20

**Authors:** Dhiraj Acharya, Amber M. Paul, John F. Anderson, Faqing Huang, Fengwei Bai

**Affiliations:** 1 Department of Biological Sciences, University of Southern Mississippi, Hattiesburg, Mississippi, United States of America; 2 Department of Entomology, Connecticut Agricultural Experiment Station, New Haven, Connecticut, United States of America; 3 Department of Chemistry and Biochemistry, University of Southern Mississippi, Hattiesburg, Mississippi, United States of America; Johns Hopkins Bloomberg School of Public Health, UNITED STATES

## Abstract

Chikungunya virus (CHIKV) is a mosquito-transmitted alphavirus that can cause fever and chronic arthritis in humans. CHIKV that is generated in mosquito or mammalian cells differs in glycosylation patterns of viral proteins, which may affect its replication and virulence. Herein, we compare replication, pathogenicity, and receptor binding of CHIKV generated in Vero cells (mammal) or C6/36 cells (mosquito) through a single passage. We demonstrate that mosquito cell-derived CHIKV (CHIKV_mos_) has slower replication than mammalian cell-derived CHIKV (CHIKV_vero_), when tested in both human and murine cell lines. Consistent with this, CHIKV_mos_ infection in both cell lines produce less cytopathic effects and reduced antiviral responses. In addition, infection in mice show that CHIKV_mos_ produces a lower level of viremia and less severe footpad swelling when compared with CHIKV_vero_. Interestingly, CHIKV_mos_ has impaired ability to bind to glycosaminoglycan (GAG) receptors on mammalian cells. However, sequencing analysis shows that this impairment is not due to a mutation in the CHIKV *E2* gene, which encodes for the viral receptor binding protein. Moreover, CHIKV_mos_ progenies can regain GAG receptor binding capability and can replicate similarly to CHIKV_vero_ after a single passage in mammalian cells. Furthermore, CHIKV_vero_ and CHIKV_mos_ no longer differ in replication when N-glycosylation of viral proteins was inhibited by growing these viruses in the presence of tunicamycin. Collectively, these results suggest that N-glycosylation of viral proteins within mosquito cells can result in loss of GAG receptor binding capability of CHIKV and reduction of its infectivity in mammalian cells.

## Author Summary

Chikungunya virus (CHIKV) is a chronic arthritis-causing pathogen in humans, for which no licensed vaccine or specific antiviral drug is currently available. Due to the global spread of its mosquito vectors, CHIKV is now becoming a public health threat worldwide. CHIKV can replicate in both mammalian and mosquito cells, however it does not cause apparent damage to mosquito cells, yet it rapidly kills mammalian cells within a day after infection. In addition, mosquito and mammalian cells have different mechanism of protein glycosylation, which can result in different glycan structures of viral glycoproteins. In this study, we report that mosquito cell-generated CHIKV has lower infectivity in cell culture and causes less severe disease in mice, when compared to mammalian cell-generated CHIKV. We demonstrate that only mammalian cell-generated CHIKV, but not mosquito-cell generated CHIKV, binds to mammalian cell surface glycosaminoglycan receptors. Interestingly, mosquito-cell generated CHIKV can re-acquire glycosaminoglycan receptor binding capability after a single passage in mammalian cells and replicate at similar levels with mammalian cell-generated CHIKV, suggesting that passage of CHIKV in mosquito cells can reduce its infectivity.

## Introduction

Chikungunya virus (CHIKV) is a mosquito-transmitted, single-stranded RNA virus belonging to the genus *Alphavirus* of the family *Togaviridae*. In humans, CHIKV infection can cause fever, headache, maculopapular rashes, myalgia, acute joint swelling, persistent arthritis, and even life-threatening neurological or cardiovascular complications [1–5]. CHIKV was first identified in Africa in 1952 and has been endemic in the tropical Indian Ocean countries for decades [6]. In recent years, this virus has caused more widespread and noticeable outbreaks. From 2004 to 2011, approximately six million cases of CHIKV infection were reported from nearly forty countries in Africa, Asia, and Europe [6–9]. In recent years, CHIKV mosquito transmission vectors *Aedes aegypti* and *Ae*. *albopictus* have spread from tropical to temperate climates, making CHIKV an emerging pathogen within these climate zones [10,11]. In line with this, CHIKV cases have been recently reported from more than twenty-five countries in the Caribbean islands, thereby posing a potential threat to North America [12]. Unfortunately, CHIKV pathogenesis is not well understood, and there is no vaccine or specific antiviral treatment currently available for CHIKV infection [13–15].

CHIKV circulates between mammalian and mosquito hosts and this cyclical transmission may provide a suitable environment for increased viral fitness and the emergence of more pathogenic strains [16,17]. Interestingly, re-emergence of CHIKV during the 2005–2006 epidemic on Reunion Island was associated with a single point mutation in its genome, which increased CHIKV fitness within its mosquito vector *Ae*. *albopictus* [18]. Additionally, CHIKV and other alphaviruses differ in their ability to infect mammalian and mosquito cells. For example, alphaviruses can cause cytopathic effects in mammalian cells and can also shut-down the mammalian macromolecular machinery involved in cellular protein synthesis at both the transcription and translational levels [19–21]. In contrast, alphavirus infection of mosquito cells causes little to no cytopathic effects and does not affect the cellular transcription and translational processes [21–24].

Mammalian and mosquito cells have distinct cellular enzymatic systems for protein glycosylation; therefore, different post-translational processing of viral surface proteins are possible in these host cells [25], which can influence replication [26–28], pathogenesis [28,29], transmission [30], and evolution [17] of mosquito-transmitted viruses. In line with this, mammalian- and mosquito-generated arboviruses can bind to different receptors expressed on the surface of host cells. For instance, differential glycosylation of viral receptor-binding proteins in mammalian- and mosquito-generated Sindbis virus [31], West Nile virus (WNV) [32], and dengue virus [33], can affect binding of these virus to host cell receptors. Similarly, mammalian cell-generated Ross River virus (RRV), Venezuelan equine encephalitis virus (VEEV), and WNV can induce more potent interferon responses compared to their mosquito cell-generated counterparts [34,35]. However, it remains unclear whether CHIKV generation in mosquito and mammalian cells can affect its infectivity and virulence.

Glycosaminoglycans (GAGs) are highly sulfated polysaccharides that are ubiquitously expressed on the cell surface and the extracellular matrix of mammalian cells [36,37]. Many viruses including CHIKV can utilize GAGs as receptors to infect host cells [38]. However, research on the role of GAG receptor binding in CHIKV and other alphaviruses has been inconclusive. The GAG receptor binding of CHIKV and other alphaviruses can be acquired through acquisition of basic amino acids in viral receptor-binding proteins *via* mutations during their continuous passage in cell culture [36,38]. Although such dependence on GAG receptor binding increases viral infectivity *in vitro*, it can potentially decreases viral fitness *in vivo* [39]. In contrast, GAG binding properties have also been described in non-cell culture adapted alphaviruses, including a clinical strain of CHIKV [40] and a wild-type strain of eastern equine encephalitis virus (EEEV) [41], suggesting that some other mechanisms that are independent of cell culture adaptation may also control GAG binding and virulence of CHIKV and other alphaviruses. Biochemically, GAG receptors possess a negative charge that enables virus-GAG receptor interaction [40–42]. In addition, mosquito and mammalian cells have different N-glycosylation mechanisms that can generate different configurations of viral glycoproteins [43] and modulate charge dependent interaction of viruses to host cell receptors. Thus, virus generation in these different host cells can potentially influence receptor binding and infectivity of CHIKV. However, the role of mosquito- and mammalian cells on GAG binding capability of CHIKV and other alphaviruses is unclear.

Herein, we report that CHIKV generated in mammalian cells replicates more efficiently during its subsequent infection of both human and murine cells *in vitro* and is more virulent in a mouse model of CHIKV arthritis, when compared to its mosquito cell-generated counterpart. We further demonstrate that the reduced replication of mosquito cell-generated CHIKV is associated with its failure to bind to cell surface GAG receptors on mammalian cells due to differential glycosylation of viral proteins in mosquito cells.

## Materials and Methods

### Ethics statement

This study was carried out in strict accordance with the recommendations described in the Guide for the Care and Use of Laboratory Animals of the National Research Council of The National Academies. The Institutional Animal Care and Use Committee at the University of Southern Mississippi (Animal Welfare Assurance # A3851-01) reviewed and approved all the animal care and use procedures under the protocol #12041201.

### Biosafety

All *in vitro* experiments and animal studies involving CHIKV were performed by certified personnel in biosafety level 3 (BSL3) laboratories, following standard biosafety protocols approved by the University of Southern Mississippi Institutional Biosafety Committee.

### Viruses, cell culture, and chemicals

Low-passaged, Vero cell-generated CHIKV Ross strain (provided by Dr. John F. Anderson, Connecticut Agricultural Experiment Station) and LR OPY1 2006 strain (provided by Dr. Robert B. Tesh, University of Texas Medical Branch) were used as parental viral stocks in this study. Majority of experiments were performed using the Ross strain and to test strain specificity, some experiments were repeated using the LR OPY1 2006 strain. The viral stocks used in this study were prepared by a single passage of parental viruses in C6/36 (ATCC, CRL-1660) or Vero cells (ATCC, CCL-81) and designated as CHIKV_mos_ and CHIKV_vero_, respectively. All viral stocks were titered in Vero cells by a plaque-forming assay. C6/36 cells were cultured at 28°C with 5% CO_2_ in Eagle’s minimum essential media (EMEM, ATCC) supplemented with 10% fetal bovine serum (FBS). Vero cells were cultured at 37°C with 5% CO_2_ in Dulbecco’s modified Eagle’s medium (DMEM, Life Technologies) supplemented with 10% FBS. L929 (ATCC, CCL-1), NIH3T3 (ATCC, CRL-1658), Raw 264.7 (ATCC, TIB-71), human foreskin fibroblasts (HFF, ATCC, CRL-2522), human THP-1 cells (ATCC, TIB-202) and human dermal fibroblasts were cultured at 37°C with 5% CO_2_ in DMEM supplemented with 10% FBS.

To generate murine bone marrow-derived dendritic cells (mBMDC), healthy C57BL/6J mice (7 weeks old) were euthanized and bone marrow cells were recovered from both femurs. After red blood cells were lysed, the bone marrow cells were cultured in R10 medium supplemented with 10% J558L cell supernatant (as a source of granulocyte-macrophage colony-stimulating factor) at a final density of 1 × 10^6^ cells/ml at 37°C with 5% CO_2_. The medium was changed every 3 days and mBMDCs were ready for infection at day 10.

Dermatan sulfate (from porcine intestinal mucosa), chondroitin sulfate A (from bovine trachea), heparin (from porcine intestinal mucosa), sepharose CL-4B, heparin-sepharose, yeast mannan, tunicamycin, and neutral red were all purchased from Sigma.

### *In vitro* infection

Cells were plated 24 h before infection in 6-, 12- or 24-well plates to 60–80% confluence. CHIKV_mos_ or CHIKV_vero_ (MOI = 1) were added to the cells and incubated at 37°C for 1 h to allow for viral adsorption and penetration. The inoculation medium was then replaced with fresh medium to remove unadsorbed viruses. Cells were washed once with fresh medium and further incubated at 37°C with 5% CO_2_ and collected at selected time points for analysis of viral genome replication and host’s gene expression.

### Microscopy and cell viability assay

Cells were infected with CHIKV (MOI = 1 or 5) for 48 h and fixed with 4% paraformaldehyde (PFA, Electron Microscopy Science). Phase contrast images were acquired using Zeiss LSM510 META confocal imaging system (Carl Zeiss Microscopy, NY). Cell viability was quantified by toluidine blue (TB) staining, according to the previously published method [44]. Viable cells were assayed by measuring the absorbance of TB at 630 nm using a microplate reader (BIO-TEC). Percentage of viable cells was calculated after normalization to uninfected controls.

### Real time-quantitative PCR (RT-qPCR)

CHIKV infected cells were subjected to total RNA extraction using TRI-reagent (Molecular Research Center, Inc.). For RNA isolation from mouse blood samples, RNeasy mini kit (Qiagen) was used. The first-strand complementary DNA (cDNA) was synthesized using the iSCRIPT cDNA synthesis kit (Bio-Rad). RT-qPCR assays were performed in a CFX96 Real-Time system (Bio-Rad) using SYBR Green supermix (Bio-Rad). Viral RNA copy numbers were expressed as the ratio of CHIKV envelope-1 (CHIKV *E1*) to cellular *β-actin*. For cytokine RT-qPCR assay, data were presented as relative fold change (RFC) in expression by the ΔΔCT method after normalized to cellular *β-actin*. Primer sequences for *β-actin* of mice [45] and human [46] were previously described. Primers for CHIKV *E1* gene (Forward: TCC GGG AAG CTG AGA TAG AA; Reverse: ACG CCG GGT AGT TGA CTA TG), and *Ae*. *albopictus ribosomal protein 7* gene (Forward: CTC TGA CCG CTG TGT ACG AT; Reverse: CAA TGG TGG TCT GCT GGT TC) were designed using NCBI online primer designing tool. Primer sequences for host immune genes (*Ifn-α*, *Ifn-β*, *Tlr3*, *Rig-I*, *Mda-5*, *and Il-1β*) were described in a previous report [44]. All primers were synthesized by Integrated DNA Technologies.

### Plaque forming assay and viral genome copy quantification

Plaque assays were performed according to our previous report with some modifications [47]. Briefly, Vero, L929 or NIH3T3 cells were plated at 5 × 10^5^ cells/well in 6-well plates one day before infection. Virus-containing samples were added to cell monolayers to allow viral adsorption/penetration at 37°C with 5% CO_2_ for 1 h. After removing unadsorbed viruses, cells were overlaid with 1% SeaPlaque agarose (Lonza) containing medium and further incubated at 37°C with 5% CO_2_ for an additional 48 h. Plaques were counted after staining with 0.3% neutral red.

CHIKV particles in viral stocks were also quantified by RT-qPCR, as described previously [38]. Briefly, 200 μl of viral stocks were treated with 50 units (U) of RNase A (Affymetrix) for 1 h at 37°C. TRI-reagent was added to inactivate RNase and lyse viral particles, and viral RNA was isolated after adding 5 μg of tRNA as carrier. First strand cDNA synthesis and CHIKV *E1* gene quantification by RT-qPCR were performed as described above.

### Animal studies

Five week old, sex-matched C57BL/6J mice (The Jackson Laboratory) were subcutaneously inoculated on the ventral side of the right hind footpad toward the ankle with 10^5^ plaque forming units (PFUs) of CHIKV_vero_ or CHIKV_mos_ (Ross strain or LR 2006 OPY 1) in 50 μl phosphate buffer saline (PBS), or with 50 μl PBS for mock controls, according to previous publications [48–50]. Blood samples were collected in 0.5M EDTA by retro-orbital bleeding and viral RNA in these samples were quantified by RT-qPCR. The height (thickness) and breath (width) of the perimetatarsal area of inoculated feet were measured daily from day 0 to day 10 post infection (d.p.i.) by using a digital caliper (Electron Microscopy Science), and the relative increase in swelling was calculated as previously described [50]. Briefly, footpad swelling was expressed as the relative increase in swelling compared to pre-infection (*x* d.p.i.– 0 d.p.i.)/0 d.p.i.).

### Histology

Mice were euthanized and inoculated footpad tissues were collected at 6 d.p.i. and fixed overnight in 4% PFA, followed by decalcification in 10% EDTA for over 10 days. Tissues were then dehydrated, paraffin embedded, and sectioned (10 μm) with a microtome (American Optical Spencer 820), followed by staining with hematoxylin and eosin (H&E). The images were acquired using a bright-field microscope (Olympus BH2).

### Attachment assays

CHIKVs (MOI = 1 or 5) were added to cell monolayer for attachment at 4°C for 1 h followed by washing with fresh medium to remove unattached viruses. The attached viruses were quantified either by RT-qPCR or by a plaque assay in the same cells, as described above. In some experiments, media containing the unattached viruses were also collected and the unattached viruses were quantified by a plaque assay in Vero cells to confirm equal numbers of virions were added to each sample.

In addition, CHIKV attachment was analyzed by flow cytometry. CHIKV_vero_ or CHIKV_mos_ (MOI = 2.5) were added to NIH3T3 cells in triplicates in PBS supplemented with 2% FBS (staining buffer) and were incubated at 4°C for 45 min. Unbound viruses were removed by washing twice with the staining buffer and the cells were fixed with 2% PFA (Electron Microscopy Science) for 15 min at room temperature (RT). After washing, the cells were probed with mouse monoclonal anti-CHIKV antibody (Abcam) and Cy5 conjugated goat anti-mouse IgG (KPL) secondary antibody, both for 1 h at RT. The cells were then washed twice and re-suspended in the staining buffer and analysed in a BD LSRFortessa (BD Biosciences) using FACSDiva version 6.0 software (BD Biosciences).

### Virus entry assays

To assess CHIKV_vero_ and CHIKV_mos_ entry into the host cells, we blocked the endosome acidification process using a lysomotrophic agent alone or in combination with a low pH medium (pH 5.5), the latter mediated viral envelope and cytoplasmic membrane fusion, as previously described [51,52]. Briefly, infection was carried out in the medium containing 20 mM NH_4_Cl to block endosomal acidification. For the direct membrane fusion assay, viruses were allowed to attach onto cells and then immediately treated with low pH medium for 2 minutes. The internalized viruses were quantified by RT-qPCR and plaque assays.

### Blocking assays for virus attachment

To analyze virus binding to GAG receptors, we performed a GAG neutralization assay, in which viruses were pre-incubated with soluble GAGs to inhibit their attachment to cell surface GAG receptors. Briefly, viruses (2.5 x 10^6^ PFU/ml) were pre-incubated with heparin, chondroitin sulfate A or dermatan sulfate (concentration indicated in figures) in DMEM containing 2% FBS at 37°C for 1 h. The virus-GAGs mixtures were then added to cells (MOI = 1) at 4°C for 1 h to allow attachment. The unattached virus-GAGs mixtures were removed and cells were washed once with fresh culture medium. The viruses attached to cells were quantified by RT-qPCR and plaque assays. In some experiments, the effect of heparin-pretreatment on viral replication was measured at 24 hours post-infection (h.p.i.) by RT-qPCR.

To measure virus binding to lectin receptors such as DC-SIGN and L-SIGN, we performed a blocking assay in the presence of yeast mannan that disrupts interaction of viruses to cell surface lectin receptors. Briefly, cells were pretreated with different concentrations of yeast mannan for 30 minutes at room temperature. Viruses were then added to the cells (MOI = 1) and incubated at 4°C for 1 h. The viruses attached on cells were quantified by RT-qPCR.

For flow cytometric analysis of GAG neutralization, CHIKV_vero_ or CHIKV_mos_ (2.5 × 10^6^ PFU/ml) were pre-incubated with different concentrations of GAGs in DMEM containing 2% FBS at 37°C for 1 h. Virus-GAGs mixtures were added to NIH3T3 cells in PBS supplemented with 2% FBS (MOI = 2.5) and incubated at 4°C for 45 min. Cells were washed twice at 4°C to remove unbound virus and immediately fixed with 2% PFA for 15 min. Cells were then probed with anti-CHIKV antibody and analyzed by flow cytometry, as described above.

### Heparin sepharose bead binding assays

Heparin-conjugated sepharose beads or unconjugated control beads were purchased from Sigma. The beads (60 μl) were washed twice in 200 μl DMEM, and mixed with 10^5^ PFUs of CHIKV in a total of 60 μl DMEM containing 2% FBS, and incubated at 4°C for 30 min. The beads were then washed three times in DMEM containing 2% FBS and the washed solution was collected for subsequent plaque assays to quantify the unbound viruses. Viruses bound to beads were lysed in 50 μl of Laemmli sample buffer (Bio- Rad), and viral proteins were separated by 10% SDS-polyacrylamide gel electrophoresis and transferred to a nitrocellulose membrane (Bio-Rad). After blocked with 5% bovine serum albumin (BSA) for 1 h at RT, the membranes were probed with mouse monoclonal anti-CHIKV primary antibody (Abcam) at 4°C for overnight on a rocker. The membranes were then washed five times (5 min each) with Tris-buffered saline with Tween 20 (TBS-T) buffer and reacted with horseradish peroxidase conjugated goat anti-mouse IgG secondary antibody (Jackson Immunoresearch) for 1 h at RT. The membranes were then washed and developed using SuperSignal West Pico Chemiluminiscence Substrate (Thermo Scientific) and images were acquired using a ChemiDoc MP system (Bio-Rad).

### Sequence analysis

Parental CHIKV viruses (original stocks received from suppliers), single-passaged CHIKV in Vero cells (CHIKV_vero_) or mosquito cells (CHIKV_mos_), and single-passaged CHIKV_vero_ and CHIKV_mos_ in NIH3T3 cells were subjected to RNA isolation using RNeasy Mini Kit (Qiagen). cDNA was prepared using the iScript cDNA synthesis kit (Bio-Rad). Complete CHIKV *E2* gene was amplified using a Q5 high fidelity polymerase (New England Biolab). The PCR primers were used according to a previous report [38]. The PCR fragments were purified by PureLink quick PCR Purification Kit (Life Technologies) and sequenced by Functional Biolab.

### Concentration of CHIKV and protein glycosylation assays

Stocks of CHIKV (Ross strain) were prepared in Vero and C6/36 cells, UV-inactivated, and viruses were concentrated by pelleting with 20% sucrose at 28,000 rpm for 2 h in an ultracentrifuge (Beckman Coulter). Deglycosylation of viral proteins were carried out using peptide-N-glycosidase F (PNGase F, Sigma) treatment following the manufacturer’s instruction. Viral proteins were separated in a 10% Mini-PROTEAN Precast Gels (Bio-Rad) and imaged in a ChemiDoc MP system (Bio-Rad) after coomassie brilliant blue staining.

### Tunicamycin treatment assays

Tunicamycin (TM) was purchased from Sigma and dissolved (10 mg/ml) in cell culture grade dimethyl sulfoxide (DMSO, ATCC). Vero cells and C6/36 cells were plated for 24 h and infected with a 0.1 MOI of parental CHIKV (Ross strain). Viruses were allowed to adsorb and penetrate for 1 h at 37°C. After unadsorbed viruses were removed, the cells were further cultured with medium containing 0.1 μg/ml of TM or the same final concentration of DMSO (0.001%) as vehicle controls. Cell culture media were collected at 24 h for virus quantification by plaque assays and RT-qPCR assays. Virus stocks generated in Vero and C6/36 cells in the presence of TM or DMSO were used to infect NIH3T3 cells (MOI = 0.1) and viral genome replication was analyzed at 24 h by RT-qPCR.

### Statistical analysis

Data were analyzed using GraphPad Prism (version 6.0, GraphPad software) and *p* < 0.05 was considered statistically significant. Data were compared using the two-tailed student's t-test or analysis of variance (ANOVA).

## Results

### CHIKV_mos_ has a lower level of replication in murine and human cells

**Fig 1:**
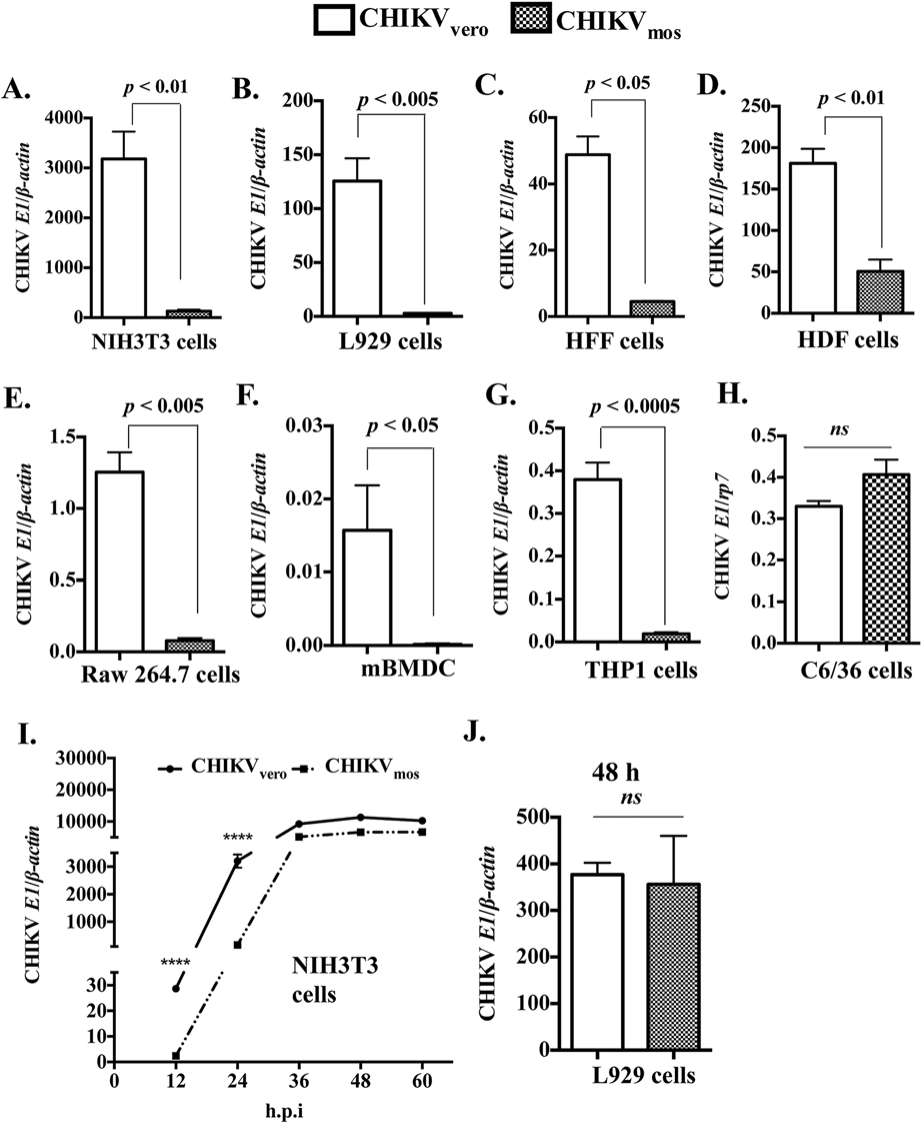
CHIKV_mos_ has reduced infectivity than CHIKV_vero_. (A-H) Indicated cells were infected with CHIKV_vero_ or CHIKV_mos_ (Ross strain, MOI = 1) for 24 h to measure gene expression of CHIKV *E1* and cellular *β-actin* by RT-qPCR. (I) NIH3T3 cells were infected with CHIKV_vero_ and CHIKV_mos_ (Ross strain, MOI = 1) for 60 h and CHIKV *E1* expression was measured at indicated time points by RT-qPCR. (J) L929 cells were infected with CHIKV_vero_ or CHIKV_mos_ (Ross strain, MOI = 1) for 48 h to measure gene expression of CHIKV *E1* by RT-qPCR. Results were shown as the ratio (mean ± SEM) copy number of CHIKV *E1* to cellular *β-actin*. *Ribosomal protein 7 (rp7*) was used as a housekeeping gene control for C6/36 cells. All data represent at least two independent experiments performed in triplicates with similar results. Replication between CHIKV_vero_ and CHIKV_mos_ were compared using student’s t test (**** denotes *p <* 0.0001, and *ns* denotes *p* ≥ 0.05).

Previous reports have suggested that passage of virus through mosquito and mammalian cells can modulate arboviral infectivity [29,31,43]. To investigate the difference between mosquito and mammalian cells generated CHIKV, we prepared CHIKV stocks (Ross strain) by infecting African green monkey (mammal) kidney cell line (Vero cells) or an *Ae*. *albopictus* (mosquito) cell line (C6/36 cells). Thus generated CHIKV stocks in Vero or C6/36 cells were titered by plaque assay in Vero cells and designed as CHIKV_vero_ and CHIKV_mos_, respectively. CHIKV replicates more efficiently in fibroblastic cells compared to hematopoietic cells [53,54], therefore we infected mouse embryonic fibroblasts (NIH3T3 cells) with CHIKV_mos_ or CHIKV_vero_ at a multiplicity of infection (MOI) of 1. At 24 h.p.i., the cells were collected for total RNA extraction and the first-strand complementary DNA (cDNA) synthesis. CHIKV envelope-1 (*E1*) gene RNA copy numbers were quantified by reverse transcription quantitative polymerase chain reaction (RT-qPCR) and cellular *β* ***-*** *actin* was used as an internal control. The RT-qPCR results showed that the level of CHIKV_mos_ replication was significantly lower (approximately 25-folds) than CHIKV_vero_ at 24 h.p.i. (Fig 1A, *p* < 0.01). In addition, we also confirmed that CHIKV_mos_ had lower replication in mouse subcutaneous fibroblasts (L929 cells, Fig 1B, *p* < 0.05), human foreskin fibroblastic cells (HFF cells, Fig 1C, *p* < 0.05) and human dermal fibroblasts (HDF cells, Fig 1D, *p* < 0.01) at 24 h.p.i. by RT-qPCR assay. To further test whether CHIKV_mos_ had lower replication over CHIKV_vero_ in cells other than fibroblasts_,_ we compared their replication in a mouse macrophage cell line (Raw 264.7 cells), primary mouse bone marrow derived dendritic cells (mBMDC), and a human monocyte cell line (THP-1). Although CHIKV replication levels were relatively lower in these immune cells when compared to fibroblasts, similar reduction of CHIKV_mos_ replication over CHIKV_vero_ was also observed in Raw 264.7 cells (Fig 1E, *p* < 0.005), mBMDC (Fig 1F, *p* < 0.05), and THP-1 cells (Fig 1G, *p* < 0.0005). In contrast to murine and human cells, both CHIKV_vero_ and CHIKV_mos_ replicated similarly when their gene copy numbers were compared in mosquito (C6/36) cells (Fig 1H).

To further study the kinetics of CHIKV_vero_ and CHIKV_mos_ replication over a longer infection period, we infected NIH3T3 cells with CHIKV_vero_ or CHIKV_mos_ (MOI = 1) and cells were collected at various time points to analyze CHIKV *E1* gene replication by RT-qPCR. In NIH3T3 cells, levels of CHIKV_mos_ replication was about 30-fold lower than CHIKV_vero_ at 12 and 24 h.p.i (Fig 1I, *p* < 0.0001), but both viruses replicated at comparable levels at the later time points (36, 48 and 60 h.p.i). Both CHIKV_vero_ and CHIKV_mos_ also replicated at comparable levels at 48 h when assayed in L929 cells (Fig 1J). These observations suggest that CHIKV_vero_ replicates more efficiently than CHIKV_mos_ in murine and human cells during the early time points. However, over the course of infection in mammalian cells, CHIKV_mos_ may gain its infectivity and replicates similarly to CHIKV_vero_.

Besides using PFU/ml as a standard titer for infection assays, we also determined viral titers by measuring viral genome copies in our viral stocks using RT-qPCR. We infected NIH3T3 cells with equal amounts of genome copies of CHIKV_vero_ and CHIKV_mos_, and compared their replication levels by RT-qPCR. Similarly, we observed a significantly lower replication of CHIKV_mos_ compared to CHIKV_vero_ (S1A Fig), which suggests that the lower replication of CHIKV_mos_ over CHIKV_vero_ was not due to the difference in unencapsidated viral genome present in our viral stocks. To test whether our results were specific to the CHIKV Ross strain, we also compared the replication levels of Vero cell-generated and C6/36 cell-generated CHIKV-LR OPY1 strain. Similarly, we observed a lower replication of C6/36 cell-generated CHIKV-LR OPY1 when NIH3T3 cells were infected (S1B Fig). Collectively, these results demonstrate that mosquito cell-generated CHIKV has reduced levels of replication in both murine and human cells during early stage of infection, when compared to Vero cell-generated CHIKV.

### CHIKV_mos_ induces moderate cytopathic effects in both murine and human cells

**Fig 2:**
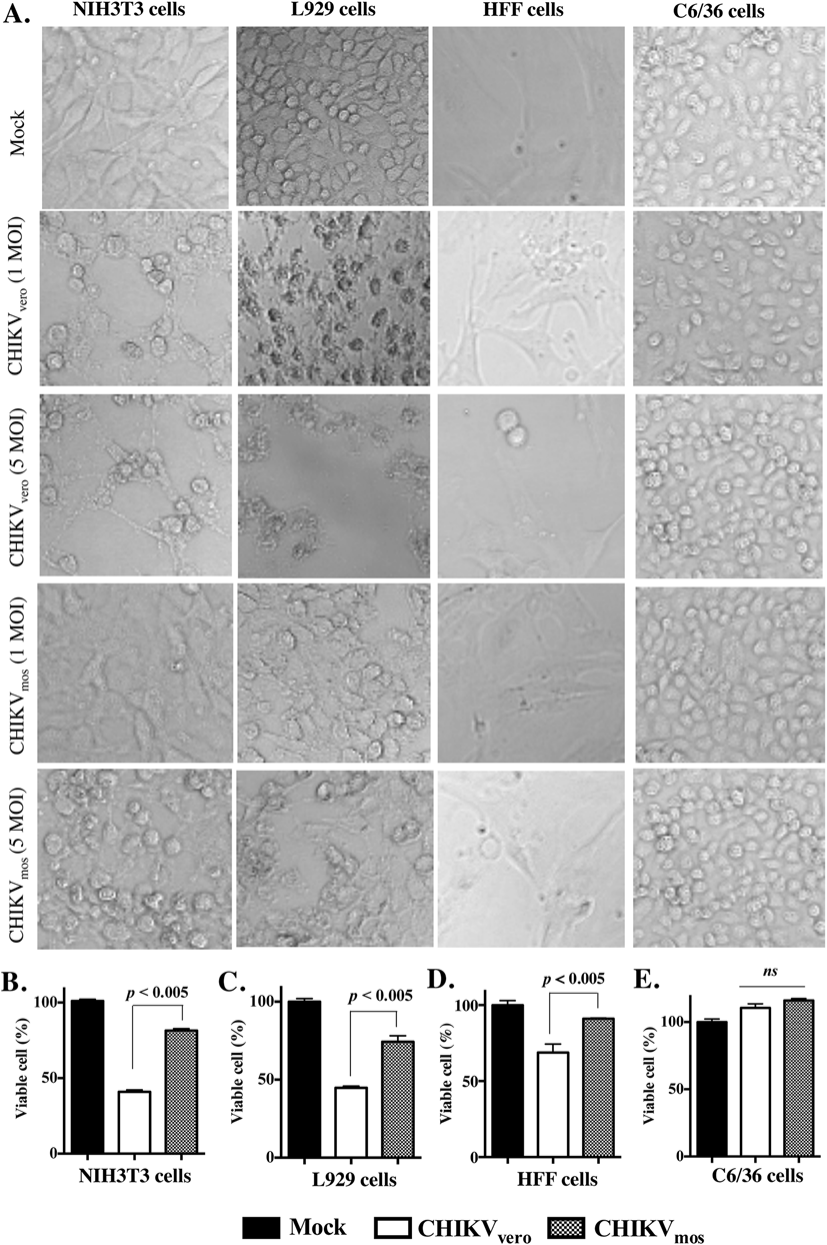
CHIKV_mos_ induces minimal cytopathic effects than CHIKV_vero_. NIH3T3, L929, HFF, and C6/36 cells were infected with CHIKV_mos_ or CHIKV_vero_ (Ross strain, MOI as indicated) for 48 h and fixed with 4% PFA after removing culture medium. (A) Phase contrast images (100X) were acquired using a Zeiss LSM510 META microscope. The cell viability data of NIH3T3 (B), L929 (C), HFF (D) and C6/36 (E) cells infected with CHIKV_mos_ or CHIKV_vero_ (Ross strain, MOI = 1) for 48 h are presented as the percentage of viable cell as determined by toluidine blue staining. The controls represent cells without viral infection (Mock). Error bars indicate mean ± SEM. Cell viability data represent two independent experiments performed in triplicates with similar results.

CHIKV infection can cause cytopathic effects and lysis of mammalian cells [21]. Since CHIKV_mos_ has a much slower replication than CHIKV_vero_ in both mouse and human cells at the early time points post infection, we expected that CHIKV_mos_ might also cause less cytopathic effects in these cells. To test this, we infected NIH3T3, L929, HFF, and C6/36 cells with CHIKV_mos_ and CHIKV_vero_ (Ross strain, MOI = 1 or 5) for 48 h, a time point when cytopathic effects were clearly visible under a microscope. The microscopy results showed that CHIKV_vero_ caused more morphological distress and cell death than CHIKV_mos_ in both human and mouse fibroblasts, but no cytopathic effect was observed in C6/36 cells (Fig 2A). This observation was further confirmed by a cell viability assay using toluidine blue staining, which showed that CHIKV_mos_ only caused moderate cytopathic effects compared to CHIKV_vero_ in NIH3T3 (Fig 2B, *p* < 0.005), L929 (Fig 2C, *p* < 0.005) and HFF cells (Fig 2D, *p* < 0.005). In contrast to murine and human cells, both CHIKV_mos_ and CHIKV_vero_ did not cause any apparent cytopathic effects in C6/36 cells (Fig 2E). To rule out the possibility that the differences in cytopathic effects were not due to Vero and mosquito cell-specific proteins that could be released in culture supernatant and might be present in our virus stocks, we examined the cytopathic effects of UV-inactivated CHIKV_vero_ and CHIKV_mos_ stocks in L929 cells. We did not observe any cytopathic effects until 72 h post-treatment (S1C Fig), suggesting that the observed cytopathic effects were specific to CHIKV_vero_ and CHIKV_mos_ infection.

### CHIKV_mos_ induces low levels of antiviral responses

**Fig 3:**
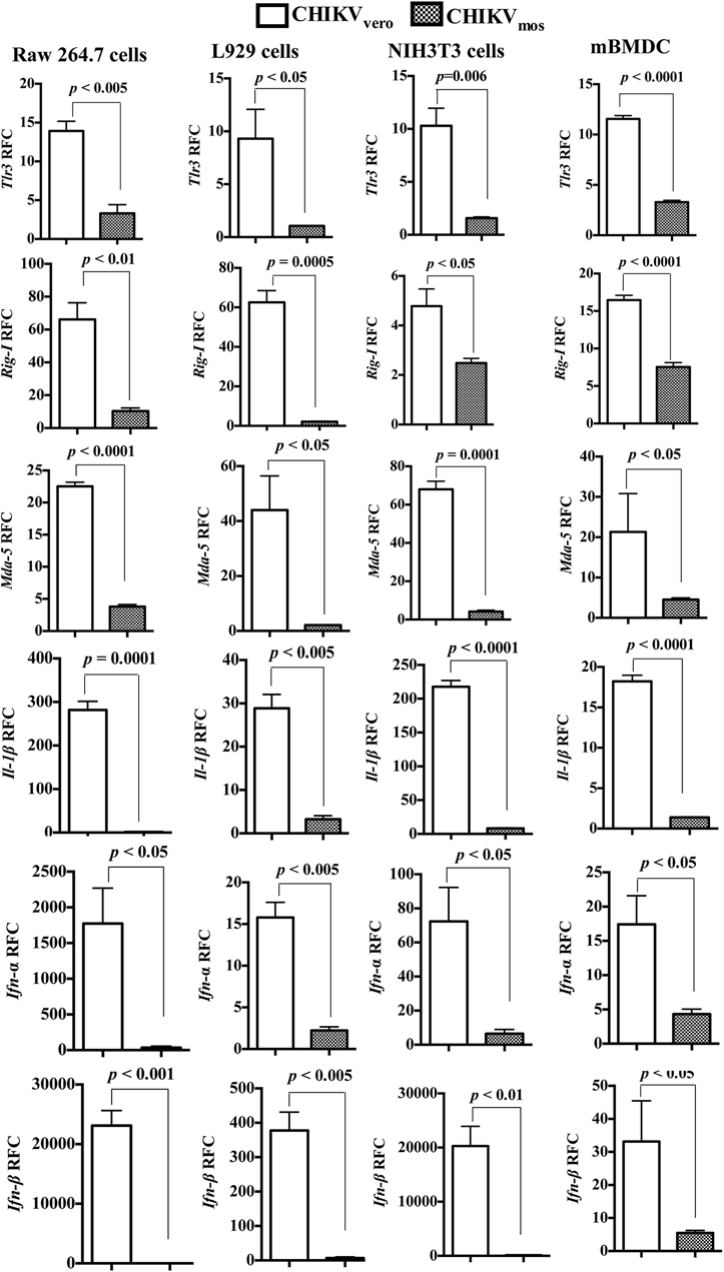
CHIKV_mos_ induces lower antiviral responses than CHIKV_vero_. Raw 264.7 cells, L929 cells, NIH3T3 cells, and mouse bone marrow derived dendritic cells (mBMDC) were infected with CHIKV_vero_ or CHIKV_mos_ (Ross strain, MOI = 1) for 24 h. Gene expressions of *Tlr3*, *Rig-I*, *Mda-5*, *Il-1β*, and type I IFNs (*Ifn-α* and *Ifn-β*) were analyzed by RT-qPCR. The gene copy numbers were normalized to cellular *β-actin* and the data are presented as relative fold changes in expression of respective gene compared to the mock-infected control designated as 1 (not indicated in figures). All data sets represents two independent experiments performed in triplicates with similar results.

Some mosquito cell-derived viruses including RRV, VEEV and WNV have been reported to exhibit enhanced infection in primary myeloid dendritic cells due to their inhibition of type I interferon production when compared to corresponding mammalian cell-derived viral preparations [34,35,55]. While our results of CHIKV_vero_ and CHIKV_mos_ were opposite to those of RRV, VEEV and WNV in terms of replication [34,35,55], we asked whether the difference in CHIKV_mos_ and CHIKV_vero_ replication was due to differential induction of cellular antiviral or inflammatory responses by these viruses. To test this, we measured the expression profiles of selected pattern recognition receptors (PRRs) and inflammatory cytokines in CHIKV_mos_ or CHIKV_vero_ (Ross strain) infected Raw 264.7, L929, NIH3T3, and mBMDC (MOI = 1) by RT-qPCR assay. In consistent with its lower replication, CHIKV_mos_ induced significantly lower levels of antiviral cytokines (*Ifn-α* and *Ifn-β*), proinflammatory cytokine (*Il-1β*), and PRRs (*Tlr3*, *Rig-I*, and *Mda-5*) in all of the tested cell types at 24 h.p.i. (Fig 3, *p* < 0.05). Difference in expression of these genes in CHIKV_vero_ and CHIKV_mos_ infected cells correspond with replication levels of these viruses in respective cells, suggesting that higher replication of CHIKV_vero_ over CHIKV_mos_ may not be due to an inhibition of antiviral or inflammatory cytokine expression by these cells. Thus, the slower replication of CHIKV_mos_ in the early stage of infection might be due to a mechanism that is independent of the host cell antiviral responses.

### CHIKV_mos_ causes less severe disease in mice

**Fig 4:**
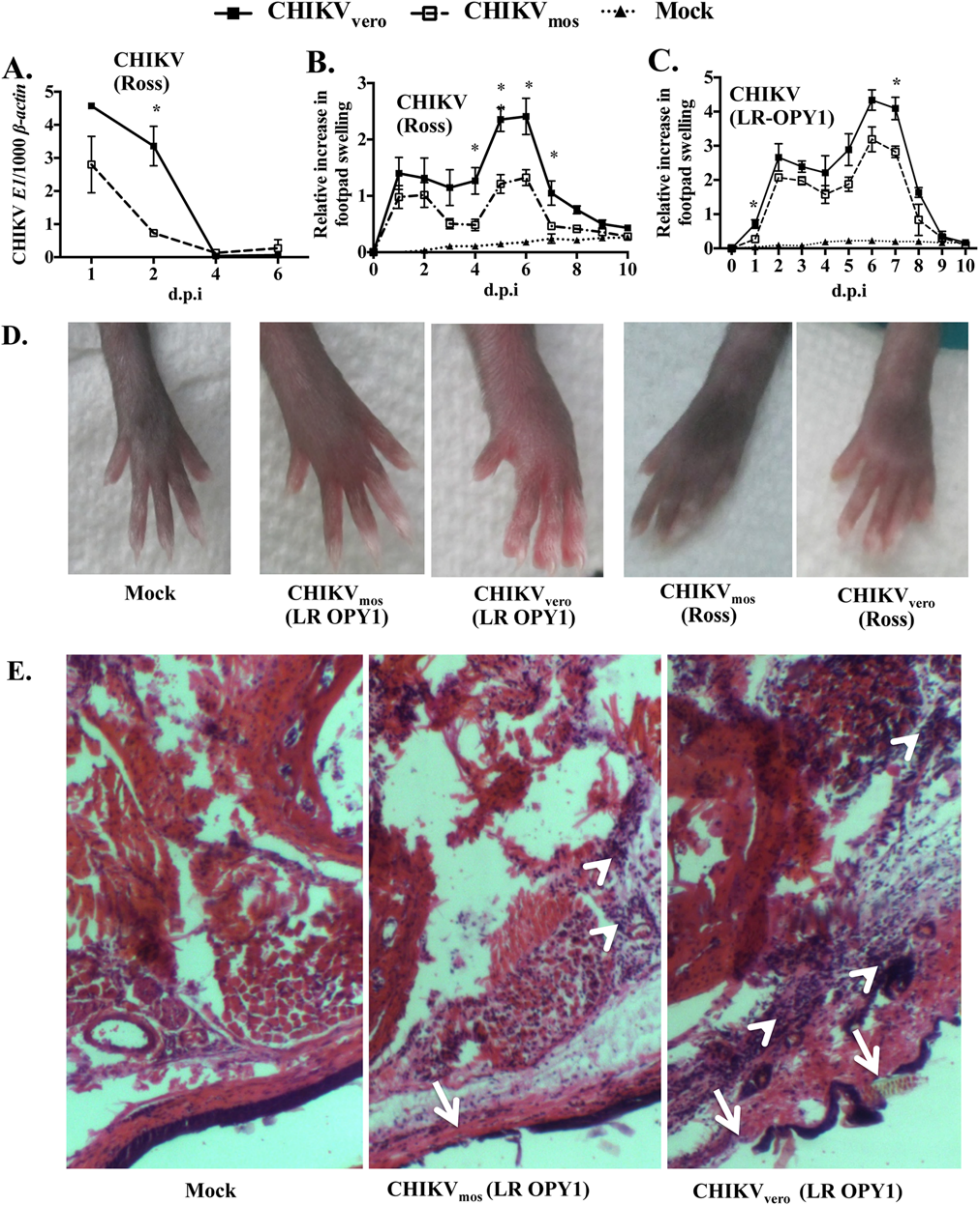
CHIKV_mos_ produces lower levels of viremia and footpad swelling in mice. Wild-type C57BL/6J mice were subcutaneously infected with 1 × 10^5^ PFUs of CHIKV_vero_, CHIKV_mos_ or mock infected with PBS and monitored daily for 10 days. (A) Viral load in blood at day 1, 2, 4 and 6-post infection (d.p.i) with CHIKV (Ross strain) is presented as ratio of CHIKV *E1* copy number per 1000 copy of cellular *β-actin*. Swelling of hind footpad (perimetatarsal area) of mice (n = 5/group) infected with CHIKV Ross (B) and LR-OPY1 (C) are presented as relative increase in swelling that were calculated by measuring height (thickness) and breadth (width) of inoculated footpad. (D) Representative image showing footpad swelling after infection with CHIKV_mos_ or CHIKV_vero_ at 6 d.p.i. (E) H&E stained histological images (100X) of foot tissue at 6 d.p.i displays subcutaneous necrosis (arrow) and infiltrated leukocytes (arrowhead). CHIKV_vero_ and CHIKV_mos_ data were compared using student’s t test (** denotes *p <* 0.005, * denotes *p <* 0.05, and *ns* denotes *p* ≥ 0.05).

Differences in *in vitro* replication of mammalian and mosquito-generated viruses may not always produce the similar clinical symptoms in a mouse model, as previously reported with WNV infection [56]. Therefore, we asked whether CHIKV_mos_ and CHIKV_vero_ also differed in their virulence *in vivo*. To test this, we infected five-week-old, sex-matched C57BL/6J mice subcutaneously *via* footpad inoculations with 1 × 10^5^ PFUs of CHIKV_mos_ or CHIKV_vero_ (Ross strain) or PBS as a vehicle control (mock), according to the previous reports [48–50]. Blood samples were collected on day 1, 2, 4 and 6 post-infection (d.p.i.) for viremia measurement by RT-qPCR, and footpad swelling was measured daily from 0 to 10 d.p.i.. CHIKV_mos_ produced lower viremia (presented as CHIKV *E1 / β-actin*) in mice over the course of infection when compared to CHIKV_vero_, which reached statistical significance at 2 d.p.i. (Fig 4A, *p* < 0.05). These results suggest that CHIKV_mos_ displays reduced infectivity in mice. Consistent with the viremia results, CHIKV_mos_ induced milder footpad swelling than CHIKV_vero_ throughout the experiment (Fig 4B and 4D). Similar results were also obtained when footpad swelling was compared in mice infected with mosquito cell- and Vero cell-generated CHIKV LR OPY1 strain (Fig 4C and 4D). To further test whether CHIKV_mos_ causes less pathology in mice compared to CHIKV_vero_, we collected inflamed footpad tissue at 6 d.p.i. and performed a histological analysis. We found that CHIKV_mos_ induced less leukocyte infiltration and limited subcutaneous necrosis in the inflamed foot when compared to CHIKV_vero_ (Fig 4E). Since type I interferons have been shown to play important roles in CHIKV pathogenesis [53,57], we also measured expression of *Ifn-*α and *Ifn-β* in the blood of CHIKV_vero_ and CHIKV_mos_ infected mice at 1, 2 and 4 d.p.i. by RT-qPCR._._ No significant difference in expression of these genes was observed between mice infected with CHIKV_vero_ and CHIKV_mos_ (S1D and S1E Fig), suggesting that lower level of swelling in mice infected with CHIKV_mos_ may not be due to difference in IFN expression, but due to lower infectivity of this virus. All these *in vivo* data suggest that CHIKV_mos_ displays lower virulence than CHIKV_vero_ in a mouse model of footpad swelling.

### CHIKV_mos_ has lower ability to attach to host cell surface receptors

**Fig 5:**
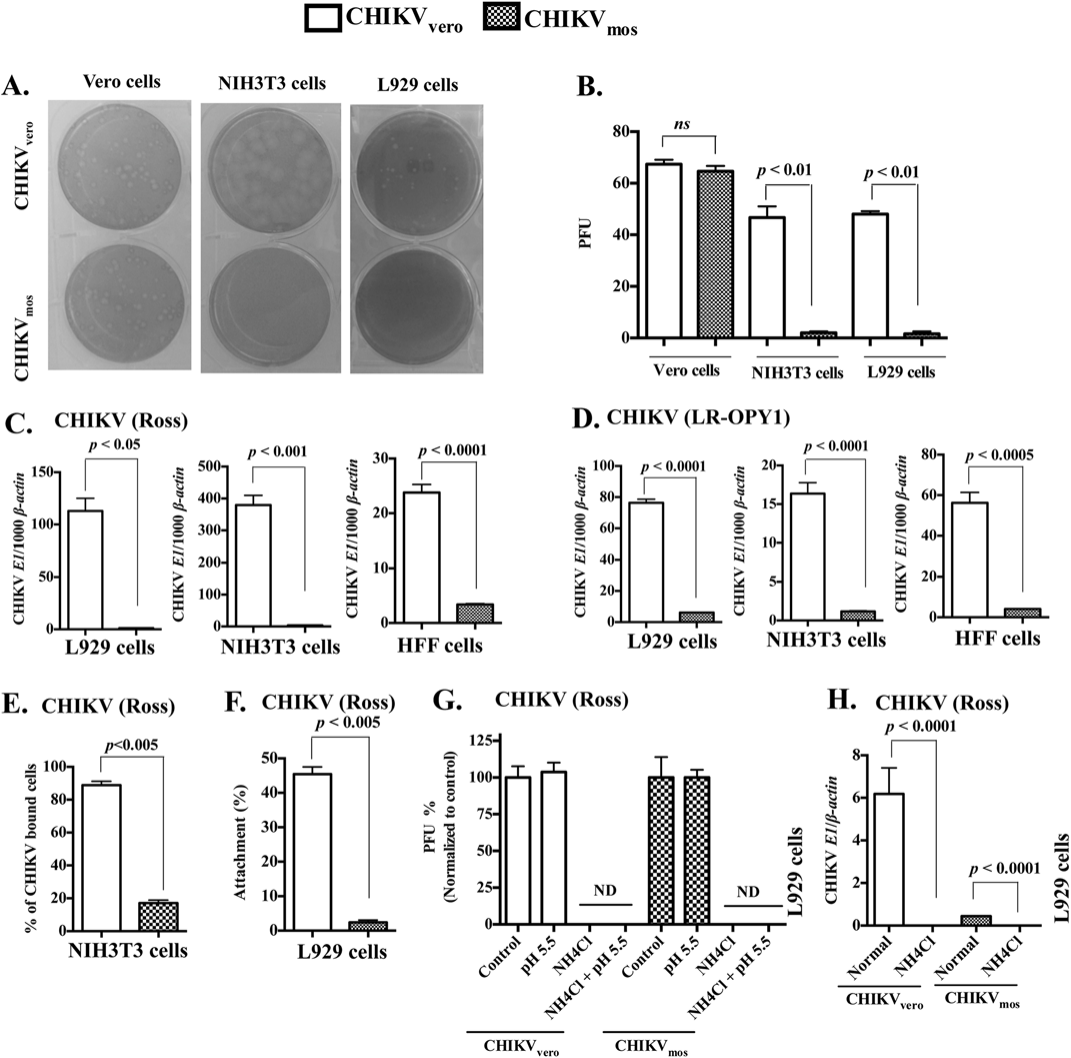
CHIKV_mos_ has reduced attachment to host cells than CHIKV_vero._ **(**A), Equal PFUs of CHIKV_vero_ or CHIKV_mos_ (Ross strain) were added to Vero cells (left), NIH3T3 cells (middle) and L929 cells (right) for plaque development. (B), Plaque counts of CHIKV_vero_ and CHIKV_mos_ in the indicated cells were quantified. (C) L929, NIH3T3 and HFF cells were inoculated with CHIKV_vero_ or CHIKV_mos_ (Ross strain, MOI = 1) at 4°C for 1 h and attached viruses were quantified by RT-qPCR and presented as the ratio of CHIKV *E1* copy number per 1000 copy of cellular *β-actin*. (D) L929, NIH3T3 and HFF cells were inoculated with CHIKV_vero_ or CHIKV_mos_ (LR OPY1, MOI = 1) at 4°C for 1 h and viruses attached to cells were quantified by RT-qPCR. (E) NIH3T3 cells were infected with CHIKV_vero_ or CHIKV_mos_ (Ross strain, MOI = 2.5) at 4°C for 45 min and the virus-bound cells were quantified by flow cytometry. (F) CHIKV_vero_ or CHIKV_mos_ (100 PFUs) were added to monolayer of L929 cells at 4°C for 1 h and both attached and unattached viruses were quantified by plaque assay to calculate percentage attachment. (G) CHIKV_vero_ or CHIKV_mos_ (Ross strain, 100 PFUs) were added to the L929 cell monolayer at 4°C for 1 h. After removing unattached virus, cells were replaced with fresh medium (control), or treated with acidic medium (pH 5.5) for 2 minutes before adding fresh medium, or replaced with NH_4_Cl (20 mM) containing media. Viruses that entered into cells were analyzed by allowing plaque development for 48 h and normalized to controls. (H) Viral RNA copies in L929 cells infected with CHIKV_vero_ or CHIKV_mos_ (Ross strain, MOI = 1) with or without NH_4_Cl (20 mM) were measured by RT-qPCR at 24 h.p.i. Data represent at least two independent experiments performed in triplicates with the similar results. ND denotes not detected, and *ns* denotes not significant (*p* ≥ 0.05).

To further dissect the mechanism by which CHIKV_mos_ has reduced replication and virulence, we next compared the plaque-forming phenotypes of CHIKV_vero_ and CHIKV_mos_ in various cells by plaque assays and counted plaques at 48 h post infection. Consistent with RT-qPCR results (Fig 1), the plaque assays showed that CHIKV_mos_ had an approximately 25-fold reduction in PFUs over CHIKV_vero_ in both NIH3T3 and L929 cells (Fig 5A and 5B, *p* < 0.01) when equal amounts of virus (~70 PFUs) were used for plaque development. In contrast to the numbers of PFUs, both CHIKV_vero_ and CHIKV_mos_ formed plaques at 48 h and no difference in plaque size was observed in all the tested cells (Fig 5A). These results suggest that the lower replication of CHIKV_mos_ in murine and human cells may be due to its reduced ability to attach or enter into these cells.

To test whether CHIKV_mos_ attaches to cell receptors at a lower affinity compared to CHIKV_vero_, we measured the attachment of CHIKV_mos_ and CHIKV_vero_ (Ross strain, MOI = 5) on L929, NIH3T3 and HFF cells. Viruses were allowed to bind to the target cells for 1 h at 4°C, a condition at which most of the viruses attach to cell surfaces but do not enter into cells [58]. Unattached viruses were removed by washing with fresh medium and the viruses attached to cells were quantified by measurement of CHIKV *E1* RNA copies by RT-qPCR. The results showed that CHIKV_mos_ had significantly reduced attachment to L929, NIH3T3 and HFF cells (Fig 5C) when compared to CHIKV_vero_. Similar results were also obtained when mosquito- and Vero cell-generated CHIKV-LR OPY1 viruses were assayed for their attachment to these cells by RT-qPCR (Fig 5D). In contrast to murine and human cells, no difference in attachment between CHIKV_vero_ and CHIKV_mos_ was observed in C6/36 cells (S1F Fig).

To further confirm lower attachment of CHIKV_mos_, we incubated NIH3T3 cells with CHIKV_vero_ or CHIKV_mos_ (Ross strain, MOI = 2.5) at 4°C for 45 min, immediately fixed the cell surface bound viruses with 4% PFA, probed these cells with anti-CHIKV monoclonal antibody, and analyzed them by flow cytometry. The results showed that CHIKV_vero_ attached to 95% of these target cells while CHIKV_mos_ attached to only 18% (Fig 5E, *p* < 0.005), which confirmed our hypothesis that CHIKV_mos_ has a reduced attachment to host cells, when compared to CHIKV_vero_. To further test whether the reduced attachment of CHIKV_mos_ correspond to its lower infectivity, the viruses attached to these cells were also visualized by plaque development. For this, we incubated 100 PFUs of CHIKV_mos_ and CHIKV_vero_ (Ross strain) with L929 cells at 4°C for 1 h and washed away unattached viruses. The attached viruses were allowed to develop plaques for 48 h in a 37°C incubator. To ensure that equal amounts of viruses were used during this experiment, unattached viral particles were also quantified by a plaque assay in Vero cells. As expected, the sum of attached and unattached virus matched between CHIKV_vero_ and CHIKV_mos_. The attachment results are expressed as percentage using the sum of attached viruses and unattached viruses as denominator (Fig 5F, *p* < 0.005). These results showed that only 2% of CHIKV_mos_, compared to 45% of CHIKV_vero_, developed plaques in L929 cells, suggesting a 22.5-fold reduction in plaque development, when viruses were allowed to attach at 4°C. These results were in agreement with the RT-qPCR and plaque assay results showing that CHIKV_mos_ had approximately 40-fold reduced replication (Fig 1C, *p* < 0.005) and approximately 23-fold reduction in plaque numbers (Fig 5A, L929 cells, *p* < 0.01) in L929 cells when compared to CHIKV_vero_. Collectively, these attachment assay results measured by RT-qPCR, flow cytometry and plaque assays, all suggest that lower replication of CHIKV_mos_ is due to its reduced attachment to the host cells.

Alphaviruses, including CHIKV, primarily use receptor-mediated endocytosis to enter into host cells, in which viruses enter the endosome through clathrin-independent endocytosis followed by a low pH dependent viral uncoating process in the endosome to gain entry into the cytoplasm [59,60]. Besides the receptor-mediated endocytosis pathway, direct viral membrane fusion with the cytoplasmic membrane has also been described as a possible mechanism that mediates the entry of some alphavirus (e.g. Semliki Forest virus) [61]. To test the possibility that whether CHIKV_vero_ or CHIKV_mos_ may use different pathways to enter into host cells, we performed plaque assays in the presence or absence of a lysomotrophic agent (NH_4_Cl) in growth media that blocks endosomal acidification and inhibits viral entry through endosomes. To test direct viral membrane fusion with cytoplasmic membrane, we induced a low pH mediated viral fusion with the plasma membrane and further cultured cells with or without NH_4_Cl. The plaque assay results showed that both CHIKV_mos_ and CHIKV_vero_ fail to develop plaques in L929 cells (Fig 5G) and Vero cells (S1G Fig) when endosomal acidification was blocked, suggesting that both types of viruses use receptor-mediated endocytosis, but not direct viral-plasma membrane fusion, to enter into cells. To further confirm that the receptor-mediated entry of CHIKV occurs through the endosome, we infected L929 cells (MOI = 1) with CHIKV_vero_ and CHIKV_mos_ in the presence or absence of NH_4_Cl and analyzed the expression of CHIKV *E1* at 24 h by RT-qPCR. These results further demonstrated that both CHIKV_vero_ and CHIKV_mos_ enter cells via the endosome pathway (Fig 5H, *p* < 0.0001).

### CHIKV_vero_, but not CHIKV_mos_, binds to mammalian cell surface glycosaminoglycan receptors

**Fig 6:**
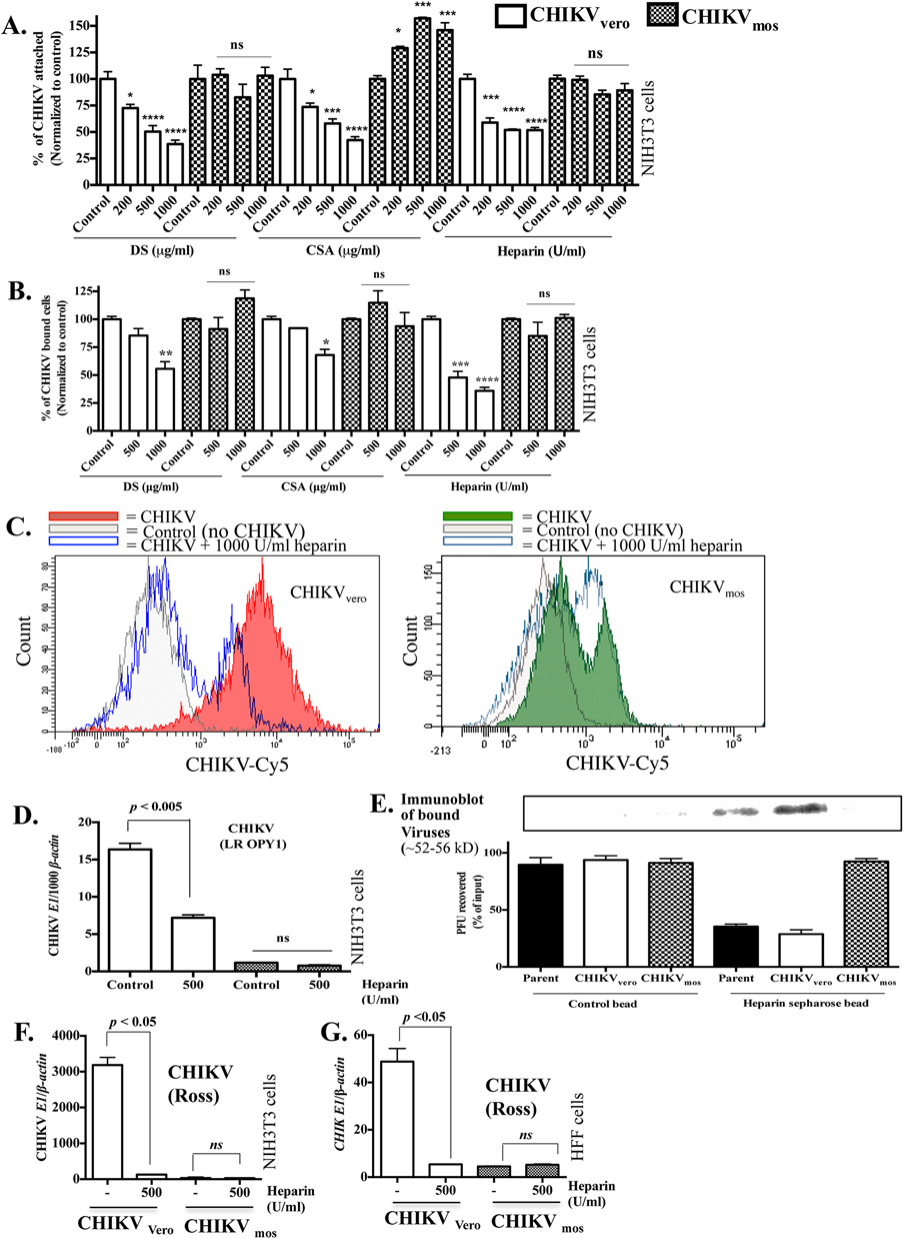
CHIKV_vero_, but not CHIKV_mos_, binds to cell surface glycosaminoglycan receptors. (A) Equal PFUs of CHIKV_vero_ or CHIKV_mos_ (Ross strain) were pre-incubated with the indicated soluble GAGs at 37°C for 1 h. The virus-GAG complexes were added to NIH3T3 cell monolayer (MOI = 1) and incubated at 4°C for 1 h and the GAGs neutralization of viral attachment was measured by RT-qPCR. Data were normalized to controls without GAG treatment. (B) Equal PFUs of CHIKV_vero_ or CHIKV_mos_ (Ross strain) were pre-incubated at 37°C for 1 h with indicated GAGs and added to NIH3T3 cells (MOI = 2.5) at 4°C for 45 min. The virus-bound cells were quantified by flow cytometry and the representative histograms are shown in (C). (D) Heparin (500U/ml) neutralization of CHIKV_vero_ and CHIKV_mos_ (LR-OPY1 strain) attachment was performed in NIH3T3 cells and measured by RT-qPCR. (E) CHIKV_vero,_ CHIKV_mos_ or their parental stock (10^5^ PFU, Ross strain) were mixed with heparin-conjugated sepharose beads or unconjugated sepharose beads (control) at 4°C for 45 min and unbound viruses recovered from beads were quantified by plaque assay in Vero cells (shown in bottom). Input viruses without beads (control) were also quantified by a plaque assay and viruses recovered from the beads were expressed as percentage of the input. Viruses bound to the respective heparin-conjugated and control beads were lysed and also analyzed by immunoblotting assays (shown in top). The effect of heparin pre-treatment (1000 U/ml) on CHIKV_vero_ and CHIKV_mos_ (Ross strain) replication was measured in NIH3T3 (F) and HFF (G) cells at 24 h by RT-qPCR. GAG treated samples were normalized to their respective controls and analyzed using a one-way ANOVA (**** denotes *p <* 0.0001, *** denotes *p <* 0.0005, ** denotes *p <* 0.005, * denotes *p <* 0.05, and *ns* denotes *p* ≥ 0.05). DS, dermatan sulfate; CSA, chondroitin sulfate A.

CHIKV E2 is the major viral protein that mediates binding of CHIKV to host cell surface receptors [62]. Although a number of cell surface receptors have been described for CHIKV and other alphaviruses [59,60], the cell surface GAG receptors have been suggested to play a role in alphavirus infectivity [40–42,63,64]. We asked whether lower attachment of CHIKV_mos_ was due to its failure to bind to the GAG receptors on host cells. To test this, we performed a GAG neutralization assay, in which we pre-incubated CHIKV_mos_ or CHIKV_vero_ (Ross strain) with a range of soluble GAGs (dermatan sulfate and heparin from porcine intestinal mucosa and chondroitin sulfate A from bovine trachea) at 37°C for 1 h. The virus-GAG mixtures were added to NIH3T3 cells and cells were further incubated at 4°C for 1 h for viral attachment. After removal of unattached viruses, the cells were washed once with fresh medium and the attached viruses were quantified by RT-qPCR. The results showed that pre-treatment with all the tested GAGs reduced CHIKV_vero_ attachment to NIH3T3 cells in a concentration-dependent manner, but did not affect the attachment of CHIKV_mos_ (Fig 6A). These results suggest that only CHIKV_vero,_ but not CHIKV_mos,_ may utilize GAG receptors to enter into cells. It has been previously shown that mosquito cell-generated alphaviruses preferentially bind to lectin receptors to enter into cells [31]. Thus, it is possible that CHIKV_mos_ uses lectin receptor to enter into cells. To test this, we performed mannan-blocking assay, in which pre-incubation of cells with yeast mannan blocks the binding of viruses to lectin receptors, such as DC-SIGN, L-SIGN and mannose receptors [31,59]. However, preincubation of both NIH3T3 and HFF cells with yeast mannan (up to 200 μg/ml) did not affect attachment of CHIKV_mos_ or CHIKV_vero_ in these cells (S1H Fig).

To further confirm GAG receptor binding of CHIKV_vero_, we also assayed GAG neutralization by flow cytometry. Similarly, we observed a concentration-dependent reduction of CHIKV_vero_ attachment to NIH3T3 cells after GAGs pre-treatment, while attachment of CHIKV_mos_ was not affected (Fig 6B and 6C). In addition, pretreatment with GAGs also reduced the plaque development of CHIKV_vero_ but not CHIKV_mos_ (S1I Fig). Similar results were also obtained when CHIKV_mos_ and CHIKV_vero_ (LR OPY1 strain) were tested for heparin neutralization in NIH3T3 cells by RT-qPCR (Fig 6D, *p* < 0.005). These results suggest that CHIKV_vero_, but not CHIKV_mos_, binds to the cell surface GAG receptors.

To further confirm differential effects of pre-incubation with soluble GAGs in CHIKV_vero_ and CHIKV_mos_ attachment to host cells, we performed a direct GAG binding assay using heparin-conjugated sepharose beads. Equal PFUs of CHIKV_vero_ or CHIKV_mos_ or their parental viruses (Ross strain, all generated in Vero cells) were incubated with heparin-conjugated sepharose beads or unconjugated control beads at 4°C for 30 min. The beads were washed three times in DMEM containing 2% FBS, and the unbound viruses in the wash solution were quantified by a plaque assay. Approximately 95% of CHIKV_vero_, CHIKV_mos_ and their parental viruses were recovered in the wash solution after incubation with the control beads (Fig 6E, bottom), suggesting that none of the tested viruses bound onto the control beads. While approximately 95% of CHIKV_mos_ were recovered in the wash solution after incubation with the heparin sepharose beads, only about 25% of CHIKV_vero_, and the parental viruses were recovered (Fig 6E, bottom), suggesting that only Vero cell-generated CHIKV (CHIKV_vero_ and their parental viruses) bound to heparin. These results were also confirmed by western blot by measuring viruses bound to the heparin sepharose and control beads (Fig 6E, top). Collectively, the results of GAG neutralization by RT-qPCR, flow cytometry, and direct heparin-conjugated sepharose bead binding assays demonstrate that only CHIKV generated in Vero cells, but not in mosquito cells, can attach to cell surface GAG receptors.

To test whether the difference in GAG receptor binding contributes to the different levels of replication of CHIKV_mos_ and CHIKV_vero_, we infected NIH3T3 and HFF cells with heparin pre-treated (1000 U/ml) or untreated CHIKV_mos_ and CHIKV_vero_ (Ross strain) at 37°C and measured viral replication at 24 h by RT-qPCR. Significant reduction in replication of CHIKV_vero_ (Fig 6G, *p* < 0.05), but not CHIKV_mos_ (Fig 6F, *p >* 0.05), was measured in both NIH3T3 cells and HFF cells (Fig 6G) after heparin pre-treatment. Of note, CHIKV_vero_ pre-incubated with heparin had comparable replication to CHIKV_mos_ in both mouse (Fig 6F) and human cells (Fig 6G), suggesting that the GAG receptor binding of CHIKV_vero_ contributes to its higher replication, when compared to CHIKV_mos_.

### Mosquito cells eliminate GAG receptor binding of CHIKV

**Fig 7:**
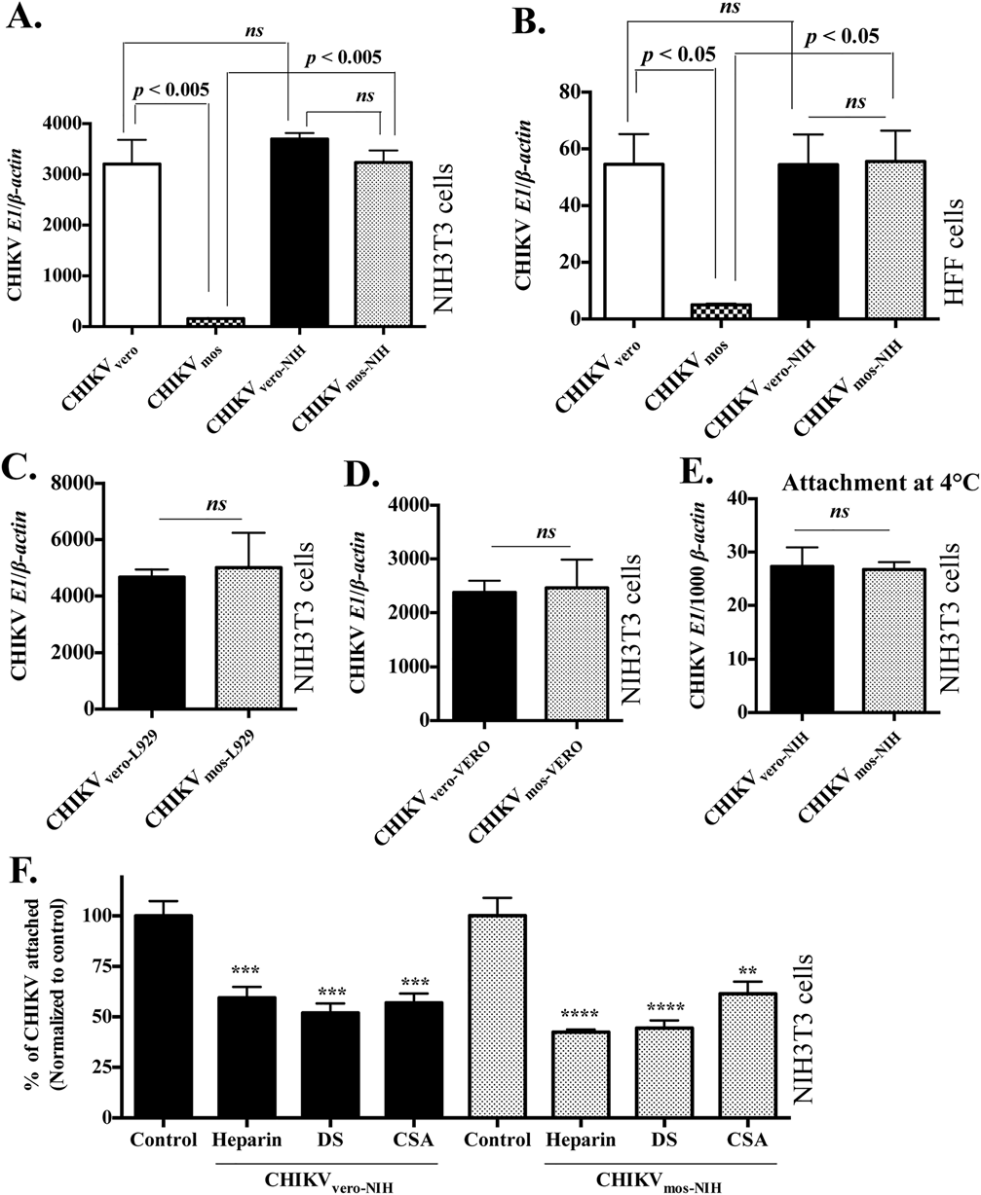
CHIKV_mos_ infectivity can be enhanced after replication in mammalian cells. The viral stocks of CHIKV_vero-NIH3T3_ and CHIKV_mos-NIH3T3_ were prepared after CHIKV_vero_ or CHIKV_mos_ (Ross strain, MOI = 1) replicated in NIH3T3 for 48 h. (A) NIH3T3 and HFF cells (B) were infected with CHIKV_vero_, CHIKV_mos_, CHIKV_vero-NIH3T3_ and CHIKV_mos-NIH3T3_ (MOI = 1), and viral RNA copy numbers were measured at 24 h by RT-qPCR. Data represents ratio of copy number of CHIKV *E1* to *β-actin*. (C) NIH3T3 cells were infected with CHIKV_vero-L929_ and CHIKV_mos-L929_ (MOI = 1), and viral RNA copy numbers were measured by RT-qPCR at 24 h. (D) NIH3T3 cells were infected with CHIKV_vero-VERO_ and CHIKV_mos-VERO_ (MOI = 1), and viral RNA copy numbers were measured at 24 h by RT-qPCR. (E) Attachment of CHIKV_vero-NIH_ and CHIKV_mos-NIH_ (MOI = 1) were performed in NIH3T3 cells at 4°C for 1 h and attached viruses were measured by RT-qPCR. (F) GAGs neutralization of CHIKV_vero-NIH3T3_ and CHIKV_mos-NIH3T3_ attachment was performed with heparin (1000 U/mL), chondroitin sulfate A (CSA, 1000 μg/mL) and dermatan sulfate (DS, 1000 μg/mL) in NIH3T3 cells, and the attached viruses were quantified by RT-qPCR and normalized to the respective untreated controls. All data sets represent two independent experiments performed in triplicates. GAG-treated samples were compared to the respective controls (without GAGs) and analyzed using a one-way ANOVA (**** denotes *p <* 0.0001, *** denotes *p <* 0.0005, ** denotes *p <* 0.005, and *ns* denotes *p* ≥ 0.05).

CHIKV E2 is the receptor binding protein of CHIKV. It has been suggested that charge-dependent interaction between the conserved basic amino acid residues in the alphavirus E2 domains and the negatively charged GAG receptors on mammalian host cell surface facilitate viral attachment [16,60]. In line with this, GAG receptor binding has been previously reported in cell-culture adapted alphaviruses, including CHIKV, whereby continuous cell-culture passages of virus result in acquisition of basic amino acid(s) in the viral E2 glycoprotein *via* mutations [38,64]. To test the possibility of potential mutations that could be acquired during CHIKV_vero_ and CHIKV_mos_ generation through a single passage, we sequenced the receptor binding protein-encoding gene *E2* of CHIKV_vero_, CHIKV_mos,_ and their parental viral stocks (both Ross and LR-OPY1 strains). The sequence alignment showed that the *E2* gene sequences were identical among CHIKV_vero_, CHIKV_mos_, and their parental stock within each strain (S2 Fig), which suggested no mutations were acquired in the *E2* gene of our CHIKV_vero_ and CHIKV_mos_ stocks after a single cell passage. In addition, the heparin-sepharose bead assays showed that the parental viruses, also generated in Vero cells, bound to soluble GAGs (Fig 6E), which indicated that Vero cell-generated CHIKV lost its GAG binding capability after a single passage in mosquito cells. To further confirm this, we generated CHIKV_mos-NIH_ and CHIKV_vero-NIH_ by growing CHIKV_mos_ and CHIKV_vero_ (Ross strain) in NIH3T3 cells for 48 h (single passage) and compared their replication levels in NIH3T3 and HFF cells at 24 h.p.i. by RT-qPCR. The results showed that both CHIKV_mos-NIH_ and CHIKV_vero-NIH_ produced a comparable amount of viral RNA and replicated similarly to CHIKV_vero_ in NIH3T3 cells (Fig 7A) and HFF cells (Fig 7B). We similarly passaged CHIKV_mos_ and CHIKV_vero_ once for 48 h in L929 to generate CHIKV_mos-L929_ and CHIKV_vero-L929_, or in Vero cells to generate CHIKV_mos-VERO_ and CHIKV_vero-VERO._ No differences in replication were observed when CHIKV_mos_ and CHIKV_vero_ passaged once in L929 cells (Fig 7C) or in Vero cells (Fig 7D) were used to infect NIH3T3 cells for 24 h. These data demonstrate that CHIKV_vero_ and CHIKV_mos_ no longer differ in replication after their single passage in mammalian cells, suggesting that mosquito cell-mediated reduction of CHIKV_mos_ replication can be regained after a single passage of CHIKV_mos_ in mammalian cells.

To further test whether CHIKV_vero_ and CHIKV_mos_ after a single passage in mammalian cells have a similar level of attachment and GAG receptor binding, we performed attachment assays and GAG receptor neutralization assays of CHIKV_mos-NIH_ and CHIKV_vero-NIH_, and measured the CHIKV *E1* gene by RT-qPCR, as described above. The RT-qPCR data showed that both CHIKV_mos-NIH_ and CHIKV_vero-NIH_ had similar levels of attachment (Fig 7E). In addition, the RT-qPCR results confirmed that pre-treatment with heparin, dermatan sulfate and chondroitin sulfate A significantly inhibited attachment of both CHIKV_mos-NIH_ and CHIKV_vero-NIH_ onto NIH3T3 cells at similar levels, confirming that CHIKV_mos_ had regained GAG receptor binding after a single passage through the mammalian cells (Fig 7F). To ensure this was not due to viral mutation, we also sequenced and compared the *E2* gene in CHIKV_mos-NIH_ and CHIKV_vero-NIH_, which showed that no mutation occurred in CHIKV *E2* gene (S2 Fig). Collectively, these results indicate that a single passage within mosquito cells can reduce CHIKV infectivity by eliminating its GAG receptor binding capability.

### Mosquito cell glycosylation reduces CHIKV infectivity in mammalian cells

**Fig 8:**
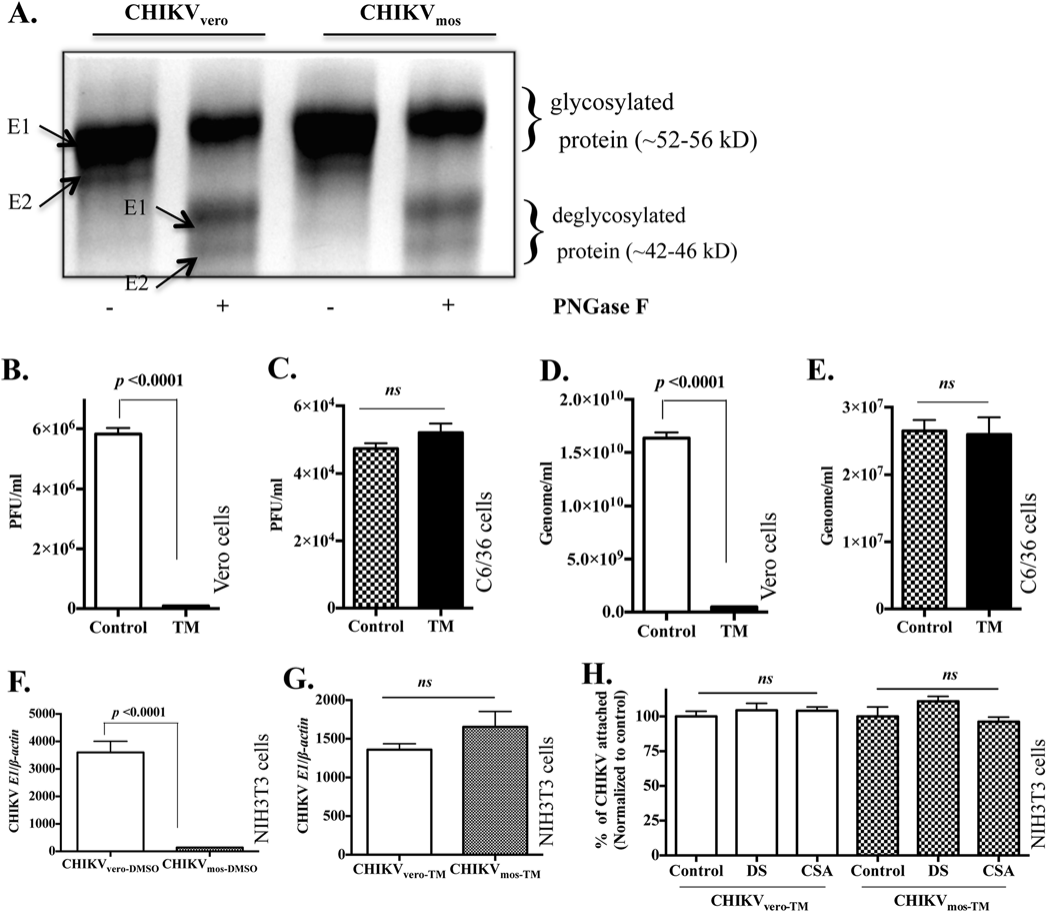
Mosquito cell-specific glycosylation reduces CHIKV infectivity. (A) SDS-PAGE image for CHIKV_vero_ and CHIKV_mos_ proteins that were treated with PNGase F to remove N-glycans from viral glycoproteins. (B-E) Vero cells or C6/36 cells were infected for 20 h with parental stocks of CHIKV (Ross) in the presence of tunicamycin (TM, 0.1 μg/ml), or with dimethyl sulfoxide (DMSO) as vehicle controls to measure infectious virus particles released in culture media by plaque-forming assay (B and C) and the viral genome copies in the culture media by RT-qPCR (D and E). NIH3T3 cells were infected with 0.1 MOI of CHIKV_vero-DMSO_ and CHIKV_mos-DMSO_ (F), or CHIKV_vero-TM_ and CHIKV_mos-TM_ (G), and viral replication was measured at 24 h by RT-qPCR. (H) GAGs neutralization of CHIKV_vero-TM_ and CHIKV_mos-TM_ binding was performed with chondroitin sulfate A (CSA, 500 μg/mL) and dermatan sulfate (DS, 500 μg/mL) in NIH3T3 cells, and the attached viruses were quantified by RT-qPCR. A two-tailed student’s t-test was used for statistical analysis. Error bars indicate mean ± SEM. *ns* denotes not significant (*p* ≥ 0.05).

Mosquito and mammalian cells use different cellular enzymes for N-glycosylation of proteins, and generate different carbohydrate residues in viral glycoproteins that influence receptor binding and virulence of viruses [31,43,65]. CHIKV E2, the receptor binding protein, is comprised of 423 amino acids with two putative N-linked glycosylation sites at positions 263 and 345 [66]. Therefore, we hypothesized that differential glycosylation of CHIKV receptor binding protein in mammalian and mosquito cells might lead to differential GAG receptor binding and therefore influence replication of CHIKV_vero_ and CHIKV_mos_. To confirm that N-glycosylation occurs in CHIKV proteins, we treated CHIKV_vero_ and CHIKV_mos_ with PNGase F, an enzyme that removes N-linked glycan from glycoproteins [67], and analyzed the viral proteins by SDS-PAGE. We observed shifts in protein bands in the gel after PNGase treatment (Fig 8A), suggesting that both E1 and E2 proteins are glycosylated in CHIKV_vero_ and CHIKV_mos_. However, the composition of glycan at these glycosylation sites differs because the post-translational modifications within insect cells generate high-mannose oligosaccharides at all glycosylation sites of viral proteins, while such modifications in vertebrate cells generate both complex and high-mannose carbohydrates chains at similar glycosylation sites [43,65,68].

To test whether removal of N-glycosylation from CHIKV_vero_ and CHIKV_mos_ affect replication of these viruses, we infected C6/36 and Vero cells for 20 h with the parental stocks of CHIKV (Ross strain) in the presence of tunicamycin (TM, 0.1μg/ml), an antibiotic that specifically inhibits N-glycosylation of proteins [26], or with DMSO as a vehicle control. We analyzed the effect of TM on virus production in mosquito cells by plaque assays and RT-qPCR. Treatment with TM reduced infectious viral particle production in Vero cells (Fig 8B, *p* < 0.001), but not in C6/36 cells (Fig 8C), when measured by plaque assay. Similar to the plaque assay results, genome quantification in culture media by RT-qPCR also showed that TM treatment similarly reduced CHIKV production from Vero cells (Fig 8D, *p* < 0.001), but not in C6/36 cells (Fig 8E). In addition, CHIKV generated in C6/36 and Vero cells in the presence of DMSO or TM has comparable genome to PFU ratio, suggesting that our observations are not due to presence of defective viral particles between groups. These results suggest that N-glycosylation of CHIKV protein can influence its replication in mammalian cells but not in mosquito cells.

We further compared the replication of non-glycosylated CHIKV_vero_ and CHIKV_mos_ that were generated after TM treatment. For this, we infected NIH3T3 cells and measured viral RNA at 24 h by RT-qPCR. As expected, CHIKV_mos_ has approximately 30-fold lower replication than CHIKV_vero_ when these viruses are generated in respective cells treated with DMSO (designated as CHIKV_vero-DMSO_ and CHIKV_mos-DMSO_, respectively) as vehicle controls (Fig 8F, *p* < 0.001). Interestingly, CHIKV_vero_ and CHIKV_mos_ generated in the presence of TM (designated as CHIKV_vero-TM_ and CHIKV_mos-TM_, respectively) no longer differed in their replication in NIH3T3 cells at 24 h (Fig 8G). To further test whether CHIKV_vero-TM_ and CHIKV_mos-TM_ differ in GAG receptor binding, we performed GAG receptor neutralization assays of these viruses by RT-qPCR as described above. The RT-qPCR data showed that GAGs pre-treatment did not inhibit binding of both CHIKV_mos-TM_ and CHIKV_vero-TM_ to NIH3T3 cells (Fig 7H). These results collectively indicate that mosquito cell-specific N-glycosylation of viral protein does not favor binding of CHIKV to cell surface GAG receptor and reduces its infectivity in murine and human cells.

## Discussion

Since viruses utilize host cell enzymatic systems to replicate their genome and synthesize functional proteins, viral infection may interrupt normal cellular functions and cause death of the host cells. However, mosquito-transmitted viruses replicate efficiently in mosquito cells, but do not cause apparent cellular damage to these cells [22,24]. Therefore, viruses may have adapted a mechanism to minimize their harmful effects within mosquito cells while replicating in mosquito vectors. This may be a viral fitness strategy, whereby a virus can efficiently replicate and generate a high titer in mosquito vectors, which is necessary for its efficient transmission to mammalian hosts. Since viral replication uses mammalian and mosquito cellular enzymatic systems to modify viral structural components [43,69], it could be possible that this fitness advantage developed in mosquito cells may reduce viral infectivity in mammalian cells [70]. To test whether mosquito or mammalian cell passages can influence CHIKV infectivity, we compared the replication levels of CHIKV_mos_ and CHIKV_vero_ generated respectively in C6/36 (mosquito, CHIKV_mos_) or Vero (mammal, CHIKV_vero_) cells through a single passage. We observed that CHIKV_mos_ had a significantly lower replication than CHIKV_vero_ in both human and murine cells, and it also induced moderate cytopathic effects and lower antiviral immune responses. In contrast, both CHIKV_mos_ and CHIKV_vero_ replicated similarly in C6/36 cells and did not caused apparent cytopathic effects to these cells (for up to 72 h.p.i.). In a mouse model of CHIKV-induced footpad swelling, CHIKV_mos_ caused lower viremia, footpad swelling, and milder histological profiles within the inoculated foot when compared to CHIKV_vero_. These results suggest that generation through mosquito cells reduces CHIKV replication in both human and murine cells and cause a less severe disease in mice.

Previous studies have demonstrated that arboviruses (e.g. Sindbis virus, WNV and dengue virus) generated in mammalian or mosquito cells can bind to different cell surface receptors [31–33]. In this study, CHIKV_mos_ replicated at lower levels at the early stages of infection (6 to 24 h) and also developed fewer plaques in both human and murine fibroblasts, compared to CHIKV_vero_. However, both CHIKV_vero_ and CHIKV_mos_ produced plaques at the same time point (approximately 48 h.p.i.) and no difference in plaque size was observed, suggesting only a portion of inoculated CHIKV_mos_ may initiate productive infection in these cells. Moreover, the cytokine expression profiles suggest that the higher replication of CHIKV_vero_ over CHIKV_mos_ may not be due to suppression of antiviral cytokines, but rather an intrinsic property of the viral particles generated in two different cell lines. Therefore, we hypothesized that CHIKV_mos_ might have a lower binding capability to murine and human cell surface receptors compared to CHIKV_vero,_ which may account for lower replication of CHIKV_mos_ at an early phase of infection. This hypothesis was proven by the attachment assay and viral entry assay results, which showed that CHIKV_mos_ had reduced receptor binding to murine and human cells. Although the cellular receptors for CHIKV and other alphaviruses remain elusive, mammalian cell surface glycosaminoglycan (GAG) receptors are the most extensively studied receptors for alphaviruses [64,71,72]. Interestingly, it has been previously demonstrated that a single passage of RRV (T48 strain) in C6/36 cells resulted in loss of GAG receptor-binding capability, while RRV derived from mammalian cells bound to GAG receptors [73]. Consistent with this report, our GAG receptor neutralization and heparin-sepharose bead binding assays showed that CHIKV_mos_ does not bind to GAG receptors. These results provided evidence for reduced receptor binding and infectivity of CHIKV_mos_ compared to CHIKV_vero_. In addition, we also demonstrated that GAG receptor binding of CHIKV_mos_ could be regained after a single passage in mammalian cells, indicating that mosquito cells can reduce infectivity of CHIKV by eliminating its ability to bind to GAG receptors on murine and human cells. It is likely that CHIKV_mos_ may use some other receptors to enter into cells, and acquire GAG receptor-binding capability after replication in mammalian cells, which could eventually enhance its infectivity.

Viruses that are generated in different host cells can acquire host cell-specific modifications on their structural components. For instance, during the viral budding process, enveloped viruses acquire a portion of the host cell membrane as the viral envelope membrane, resulting in variable carbohydrate and lipid compositions of these viruses, depending on the cell types in which they are generated [43,69,74]. In addition, mosquito and mammalian post-translational modifications, particularly the N-glycosylation, uses different cellular enzymes to modify the viral glycoproteins [31,65]. Our PNGase F treatment assay results confirm that CHIKV E2, the receptor binding protein of CHIKV, has N-linked glycosylation sites. However, the composition of the carbohydrate residues at the glycosylation sites of E2 may vary depending on the host cell types used for CHIKV generation. Insect cells, including mosquitoes, are deficient in enzymes for carbohydrate synthesis, including *N*-acetylglucosaminyl-, galactosyl-, and sialyltransferases, resulting in high-mannose oligosaccharides at all glycosylation sites. In contrast, vertebrate cells can generate both complex and high-mannose carbohydrates chains at these glycosylation sites [43,65,68]. Glycosylations of Sindbis virus (SINV) E2 at positions 196 and 318 have been documented to play critical roles in receptor binding and infectivity in both cell culture and in a mouse model [28]. Thus, we hypothesized that differential glycosylation patterns in mammalian and mosquito cells may play a role in differential infectivity of CHIKV generated in these cell lines. TM is a potent inhibitor of N-glycosylation and it has been previously shown that viral stocks prepared in the presence of TM lose glycan at their glycolysation sites [26]. We demonstrated that CHIKV_vero_ and CHIKV_mos_ no longer differed in their replication when these viruses were prepared in the presence of TM, suggesting that mosquito and mammalian cell-specific glycosylation can affect the replication of CHIKV_vero_ and CHIKV_mos_ in murine and human cells. Although we could not completely exclude the possibility that mosquito cell-specific processing of other structural and nonstructural viral proteins might also influence the infectivity of CHIKV_mos_, our data provided several lines of evidence to support that mosquito cell-specific glycosylation of E2 does not favor GAG receptor binding and reduces CHIKV infectivity.

Previous studies suggested that mammalian cell-generated RRV, VEEV, and WNV induce potent IFN responses, but these viruses generated in mosquito cells inhibit IFN production and replicate more efficiently in mammalian cells *in vitro* [34,35]. It has been suggested that mosquito cell-generated WNV has higher infectivity *in vitro* [32], however, it produced significantly lower viral load in mice compared to mammalian cell-generated WNV during an early phase of infection, indicating greater infectivity of mammalian cell-generated WNV *in vivo* [56]. We found that mammalian (Vero) cell-generated CHIKV possessed greater infectivity in both *in vitro* and *in vivo* conditions. It is possible that the lack of GAG receptor binding of mosquito-generated CHIKV may favor its replication in mosquito cells, however this can potentially reduce its infectivity in mammalian cells. This hypothesis is also supported by a recent report, which showed that RRV had increased fitness in mosquito cells but produced less severe disease in a mouse model [70].

The role of GAG receptors in viral pathogenesis has been extensively studied in human immunodeficiency virus [75], herpes simplex virus [76], Echovirus [77], and human papillomavirus [78]. However, the role of GAG receptor binding in alphavirus pathogenicity remains controversial. Several reports document that cell-culture adapted alphaviruses (about 10–20 generations) can acquire GAG receptor binding capability *in vitro* via mutations in the *E2* gene that leads to acquisition of basic amino acids [38,64,71,72]. Such GAG receptor dependence, often described as a cell-culture adapted property, has been suggested to reduce viral pathogenicity *in vivo*, presumably because of rapid clearance of these viruses from circulation, as shown in VEEV [39] and SFV [79]. In contrast, GAG receptor binding of cell-culture adapted SINV has been shown to enhance its infectivity and neurovirulence in mice [42]. It has been believed that wild-type (non cell-culture adapted) alphaviruses generally do not use heparan sulfate (HS) receptors, a prototype of GAG receptors, for host cell attachment but depend on other cell surface receptors including DC-SIGN, L-SIGN, and C-type lectin molecules [31,59]. However, it has been demonstrated that wild-type isolates of eastern equine encephalomyelitis virus (EEEV) and VEEV that bound to GAG receptors were neurovirulent in mice [41,80]. Unlike other cell culture adapted alphaviruses, the GAG receptor binding capability of wild-type EEEV does not occur through acquisition of additional basic amino acids in E2 [41]. Similarly, GAG receptor binding property has been also described in a non cell-culture adapted clinical CHIKV strain, which also occurred independent of additional basic amino acids acquisition in E2 [40]. However, a non cell-culture adapted CHIKV LR strain does not bind to GAG receptors [57], suggesting that GAG receptor binding may not be a property of all wild-type CHIKV strains. Herein, we report that mosquito cell-generated CHIKV does not bind on GAG receptors when compared to Vero cell generated counterpart, but it can acquire GAG receptors binding capability after a single passage in mammalian cells, such as NIH3T3 and L929 cells. CHIKV *E2* sequencing results further confirmed that the GAG binding ability of mammalian cell-generated CHIKV was not due to mutation. It is noteworthy that we did not detect any previously published E2 amino acids in our CHIKV strains that were described to facilitate GAGs binding of the tissue-culture adapted CHIKV strain [38], suggesting that GAG receptor binding in our mammalian cell generated CHIKV does not reflect cell-culture adaptation and occurs without acquisition of basic amino acids. Thus, our results support the notion that the GAG receptor binding capability in CHIKV and other alphavirus can also be acquired through mechanisms independent of basic amino acid acquisition in E2 and such GAG binding capability can also enhance CHIKV infectivity both *in vitro* and *in vivo*.

Attenuation of viruses through a continuous cell-culture passage has been used as a strategy to develop live-attenuated arboviral vaccines [81–84]. Live-attenuated yellow fever virus vaccine (17D strain) is one of the most successful vaccines generated through this approach. Unfortunately, uses of this approach in an attempt to generate similar vaccines to other viruses were not successful. Although the detailed molecular mechanisms for attenuation of 17D vaccine strain remain elusive, it has been proposed that viral dependence on GAG receptor binding, particularly HS receptors, which can be acquired during continuous cell-culture passage, might play a role [82,84,85]. Recently, GAG-receptor binding CHIKV strains generated through continuous cell-culture passage has been evaluated as potential vaccine candidates [38,86,87]. Although GAG receptor dependence has been believed to attenuate alphaviruses *in vivo*, some of these CHIKV vaccine strains have been shown to be pathogenic [38]. For example, CHIKV vaccine strain (181/25) has been reported to cause transient arthralgia in clinical trials [88]. Although the selection of GAG receptor dependence has been proposed as a vaccine developmental strategy, accumulating evidence [38,40,41,89] suggests that other mechanisms are also possible in attenuation of cell-culture adapted arboviruses. Therefore, strategies to generate and/or evaluate CHIKV and other arboviral vaccine candidates should not be solely based on selection of GAG receptor dependence. In addition, viruses may have different affinity of GAG receptor binding or they may bind to different types of GAG receptors that may affect GAG receptor-dependent CHIKV infectivity *in vitro* and *in vivo*, which requires further investigation.

The mechanisms by which mosquito-transmitted viruses cause minimal or no damage to mosquito vectors, yet can cause cell death and diseases in mammalian hosts, are not well understood. The cellular machinery of mosquitos, which prevents CHIKV to bind to GAG receptors, may favor viral replication in mosquito cells without causing significant cytopathic effects. Our observation of reduced infectivity of mosquito-generated CHIKV puts forward an intriguing question of how CHIKV manages to infect hosts during natural infection, when viruses are inoculated into human skin by a mosquito bite. It has been previously reported that the mosquito saliva facilitates pathogenicity of some arboviruses, including CHIKV, La Crosse, dengue, and WNV by inducing CD4^+^ T helper-2 (Th2) cell dominant anti-inflammatory responses [90–95]. It is likely that CHIKV may take advantage of mosquito salivary proteins to initiate early infection in humans. A recent report showed that an *Ae*. *aegypti* saliva serine protease enhanced dengue virus infectivity by increasing the attachment of viruses to HS proteoglycans in mammalian hosts [96], suggesting that the reduction in viral GAG receptor binding by mosquito cells can be compensated by mosquito saliva during natural infections. After inoculation into the human skin through a mosquito bite, the mosquito cell-derived CHIKV may use alternative receptors to initiate a low level of infection in human cells. During the course of infection, CHIKV replicates using human cellular machinery and acquires GAG receptor binding, resulting in its enhanced infectivity.

In conclusion, this study demonstrates that the infectivity of CHIKV is reduced when generated in mosquito cells. We show that mosquito cell-generated CHIKV does not bind to GAG receptors and has reduced attachment to mammalian cells. Furthermore, we provide several lines of evidence to support that N-glycosylation within mosquito cells may lower infectivity of CHIKV by removing its ability to bind GAG receptors on murine and human cells. This new understanding of how mosquito and mammalian host cells alter CHIKV receptor binding and infectivity may help with the development of effective therapeutics or vaccines against CHIKV and other mosquito-transmitted viruses.

## Supporting Information

S1 Fig Supplemental figures. **(A)** NIH3T3 cells were infected with CHIKV_vero_ or CHIKV_mos_ (Ross strain, 100 viral particles/cell) for 24 h and expression of CHIKV *E1* (normalized to cellular *β-actin*) was measured by RT-qPCR. **(B)** NIH3T3 cells were infected with CHIKV_vero_ and CHIKV_mos_ (LR OPY1 strain, MOI = 1) for 24 h and CHIKV *E1* expression was measured by RT-qPCR. **(C)** L929 cells were infected with UV-inactivated CHIKV_mos_ or CHIKV_vero_ (Ross strain) for 72 h and phase contrast images (100X) were acquired using a LSM510 META microscope (Zeiss). Expressions of *Ifn-α* **(D)** and *Ifn-β* **(E)** in the blood of wild-type C57BL/6J mice infected with CHIKV_vero_ and CHIKV_mos_ (Ross strain, 10^5^ PFUs) were measured in blood by RT-qPCR at day 1, 2, 4 and 6-post infection (d.p.i). **(F)** C6/36 cells were inoculated with CHIKV_vero_ or CHIKV_mos_ (Ross strain, MOI = 1) at 4°C for 1 h and the viruses attached to cells were quantified by RT-qPCR. **(H)** NIH3T3 and HFF cells were pre-incubated with the indicated concentration of yeast mannan for 1 h at room temperature. CHIKV_vero_ or CHIKV_mos_ (Ross strain, MOI = 1) were then added to the cells and further incubated at 4°C for 1 h to allow attachment of viruses. The blocking of viral attachment by yeast mannan was measured by RT-qPCR. Data were normalized to the control cells without yeast mannan treatment. **(I)** One hundred PFUs of CHIKV_vero_ or CHIKV_mos_ (Ross strain) were pre-incubated with heparin at 37°C for 1 h and then added to Vero cells at 4°C for 1 h for virus attachment. The neutralization of plaque development in Vero cells by heparin was measured by plaque assay.
(TIF)

S2 Fig Sequence alignment data. E2 glycoprotein sequences of different CHIKV stocks used in this study were shown. Ross strain (black color) and LR OPY1 strain (blue color).
(TIF)
