# Supplementary figures and images for: Loss of Glycosaminoglycan Receptor Binding after Mosquito Cell Passage Reduces Chikungunya Virus Infectivity
Source: PLoS Negl Trop Dis. 2015 Oct 20;9(10):e0004139. doi: 10.1371/journal.pntd.0004139 (PMC4615622; doi:10.1371/journal.pntd.0004139)

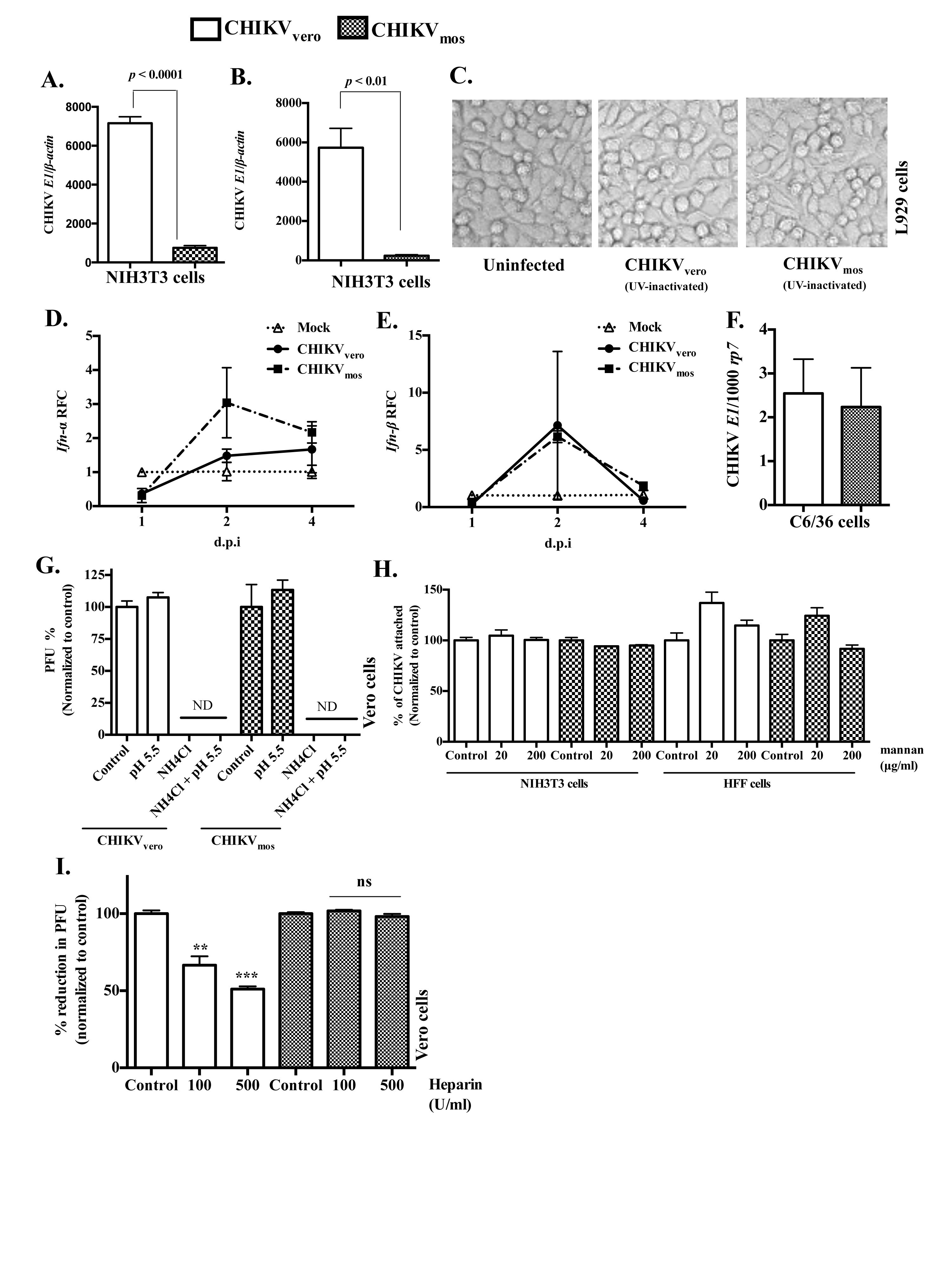

Supplement: S1 Fig — (A) NIH3T3 cells were infected with CHIKVvero or CHIKVmos (Ross strain, 100 viral particles/cell) for 24 h and expression of CHIKV E1 (normalized to cellular β-actin) was measured by RT-qPCR. (B) NIH3T3 cells were infected with CHIKVvero and CHIKVmos (LR OPY1 strain, MOI = 1) for 24 h and CHIKV E1 expression was measured by RT-qPCR. (C) L929 cells were infected with UV-inactivated CHIKVmos or CHIKVvero (Ross strain) for 72 h and phase contrast images (100X) were acquired using a LSM510 META microscope (Zeiss). Expressions of Ifn-α (D) and Ifn-β (E) in the blood of wild-type C57BL/6J mice infected with CHIKVvero and CHIKVmos (Ross strain, 105 PFUs) were measured in blood by RT-qPCR at day 1, 2, 4 and 6-post infection (d.p.i). (F) C6/36 cells were inoculated with CHIKVvero or CHIKVmos (Ross strain, MOI = 1) at 4°C for 1 h and the viruses attached to cells were quantified by RT-qPCR. (H) NIH3T3 and HFF cells were pre-incubated with the indicated concentration of yeast mannan for 1 h at room temperature. CHIKVvero or CHIKVmos (Ross strain, MOI = 1) were then added to the cells and further incubated at 4°C for 1 h to allow attachment of viruses. The blocking of viral attachment by yeast mannan was measured by RT-qPCR. Data were normalized to the control cells without yeast mannan treatment. (I) One hundred PFUs of CHIKVvero or CHIKVmos (Ross strain) were pre-incubated with heparin at 37°C for 1 h and then added to Vero cells at 4°C for 1 h for virus attachment. The neutralization of plaque development in Vero cells by heparin was measured by plaque assay. (TIF) [file pntd.0004139.s001.tif]

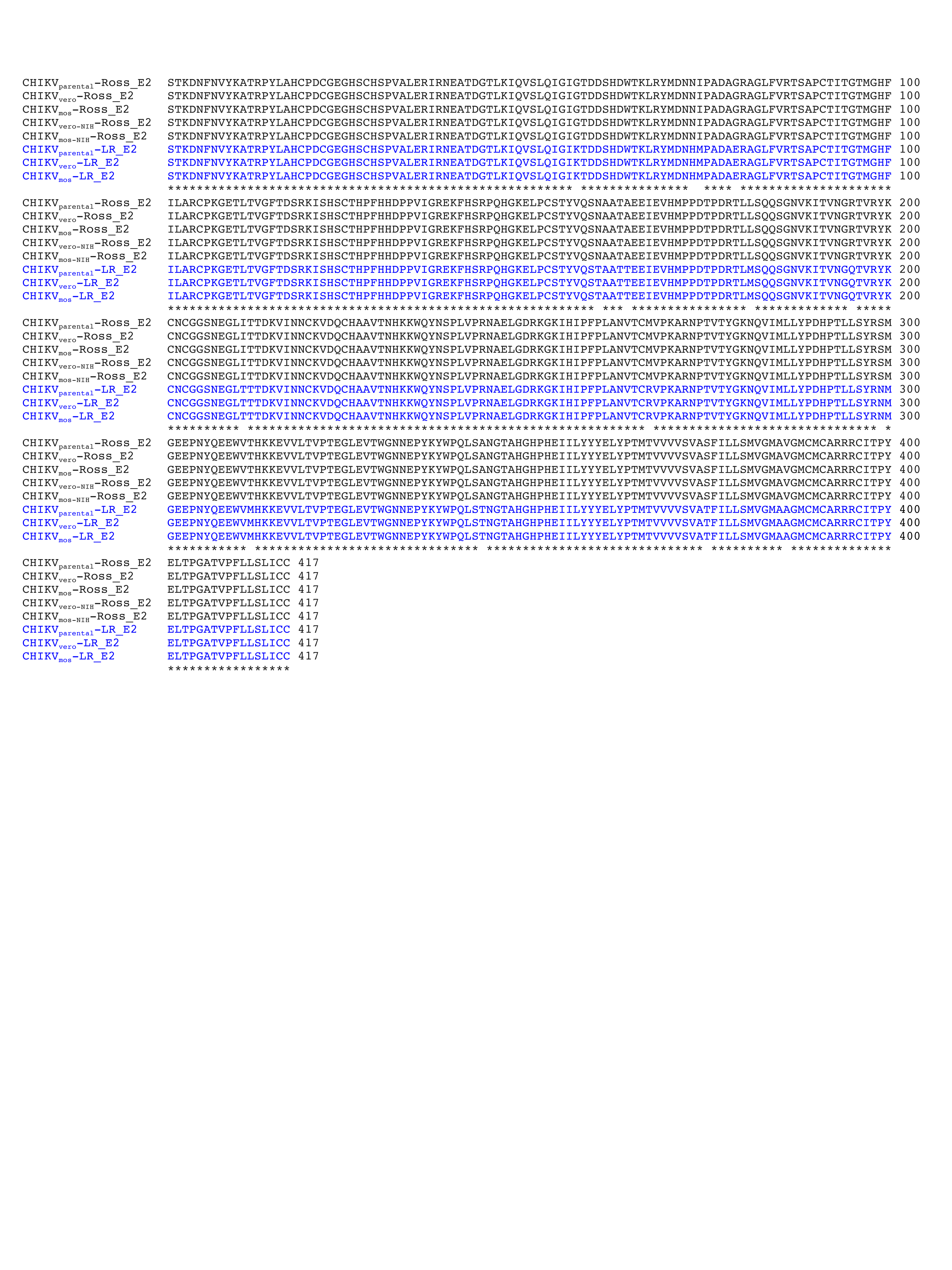

Supplement: S2 Fig — E2 glycoprotein sequences of different CHIKV stocks used in this study were shown. Ross strain (black color) and LR OPY1 strain (blue color). (TIF) [file pntd.0004139.s002.tif]
